# RNA sequencing on *Solanum lycopersicum* trichomes identifies transcription factors that activate terpene synthase promoters

**DOI:** 10.1186/1471-2164-15-402

**Published:** 2014-05-27

**Authors:** Eleni A Spyropoulou, Michel A Haring, Robert C Schuurink

**Affiliations:** Department of Plant Physiology, Swammerdam Institute for Life Sciences, University of Amsterdam, Science Park 904, Amsterdam, 1098 XH The Netherlands

**Keywords:** Tomato trichomes, Terpene biosynthesis, Transcription factor, High-throughput sequencing

## Abstract

**Background:**

Glandular trichomes are production and storage organs of specialized metabolites such as terpenes, which play a role in the plant’s defense system. The present study aimed to shed light on the regulation of terpene biosynthesis in *Solanum lycopersicum* trichomes by identification of transcription factors (TFs) that control the expression of terpene synthases.

**Results:**

A trichome transcriptome database was created with a total of 27,195 contigs that contained 743 annotated TFs. Furthermore a quantitative expression database was obtained of jasmonic acid-treated trichomes. Sixteen candidate TFs were selected for further analysis. One TF of the MYC bHLH class and one of the WRKY class were able to transiently transactivate *S. lycopersicum* terpene synthase promoters in *Nicotiana benthamiana* leaves. Strikingly, SlMYC1 was shown to act synergistically with a previously identified zinc finger-like TF, Expression of Terpenoids 1 (SlEOT1) in transactivating the *SlTPS5* promoter.

**Conclusions:**

High-throughput sequencing of tomato stem trichomes led to the discovery of two transcription factors that activated several terpene synthase promoters. Our results identified new elements of the transcriptional regulation of tomato terpene biosynthesis in trichomes, a largely unexplored field.

**Electronic supplementary material:**

The online version of this article (doi:10.1186/1471-2164-15-402) contains supplementary material, which is available to authorized users.

## Background

Specialized glandular trichomes can produce and accumulate large quantities of terpenoids, phenylpropanoids, flavonoids and alkaloids, which they can also secrete [1]. RNA sequencing in combination with metabolite profile analysis of glandular trichomes and proteomics have shed light on the biosynthesis of specialized metabolites in the trichomes of various plant species [2]. Through the production of EST libraries, micro-arrays and high-throughput sequencing of (glandular) trichome RNA, genes have been identified that are involved in the terpenoid, phenylpropanoid, alkaloid and flavonoid biosynthesis in various plant species, including tomato [3–5], sweet basil [6, 7], tobacco [8, 9], mint [10], alfalfa [11], *Artemisia annua*[12] and hop [13]. Although EST sequencing has been instrumental in the discovery of enzymes of trichome-specialized metabolism [4], next generation sequencing (NGS) can give a more in-depth picture of transcriptomes. NGS technologies (i.e. RNA sequencing) has been used for characterization of several trichome transcriptomes, for example from plants of medical importance like *Artemisia annua* (Asteraceae; [12]) or *Huperzia serrata* and *Phlegmariurus carinatus* (Huperziaceae; [14]). NGS has also been used for gene discovery, for example in combination with shotgun proteomics and metabolite analysis of tomato (*Solanum lycopersicum*) trichomes, leading to the discovery of the leaf-trichome-specific β-caryophyllene/α-humulene synthase (CAHS; [4]). NGS of trichomes RNA from wild and cultivated tomato varieties led to the discovery and characterization of various sesquiterpene synthases, providing insight into the evolution of terpene synthases [15].

Terpene biosynthesis in tomato plants is of major interest because terpenes play an important role in the plant’s defense [16–20]. The sequencing of the cultivated tomato genome has enabled the characterization of its terpene synthase (TPS) gene family [21, 22], but not much is known about the regulation of the terpenoid pathway. Transcriptional control of biosynthetic genes is a major mechanism by which secondary metabolite production is regulated [23, 24].

There are not many transcription factors (TFs) known to be involved in regulation of terpenoid pathways. ORCA3, a jasmonate-responsive APETALA2 (AP2)-domain transcription factor from *Catharanthus roseus*, has been shown to regulate expression of *Strictosidine Synthase* (*STR*) involved in terpene indole alkaloid biosynthesis [25]. Subsequently, a methyl-jasmonate (MeJA)-inducible transcription factor of the MYC family (*CrMYC2*) was shown to positively regulate *ORCA3*[26]. CrWRKY1 was identified as being involved in the root-specific accumulation of serpentine in *C. roseus* plants and as being induced by phytohormones including JA [27]. This TF appeared to negatively regulate *ORCA3* and to a lesser extend *CrMYC2*[27]. A MeJA-inducible WRKY transcription factor from *Gossypium arboreum* that regulates the sesquiterpene synthase (+)-*δ*-*cadiene synthase A* in cotton fibers was identified by Xu *et al.*[28]. Ma *et al*. [29] demonstrated that a MeJA-inducible WRKY transcription factor from *Artemisia annua* is involved in the regulation of artemisinin biosynthesis. More recently two JA-responsive AP2 family transcription factors from *A. annua* (AaERF1 and 2) were found to regulate *Amorpha-4,11-diene synthase* (*ADS*), a sesquiterpene synthase involved in the biosynthesis of artemisinin [30] whereas Lu *et al*. [31] identified AaORA, a AP2/ERF TF, that regulates several genes in the artemisinin biosynthetic pathway including *AaERF1*. Most recently, the MeJA-inducible *Arabidopsis thaliana* MYC2 transcription factor [32] was shown to regulate sesquiterpene synthases *AtTPS21* and *AtTPS11*[33].

Here, we used NGS of tomato stem trichomes as a tool for gene discovery. First, a transcript database was created from normalized cDNA, which was mined for transcription factors. Then, in order to narrow down the number of TFs potentially involved in terpene biosynthesis, an expression profiling database was created using Illumina sequencing of trichome RNAs from plants treated with or without jasmonic acid (JA), since JA is known to induce terpene emission in tomato and to regulate several terpene synthases [16, 21, 34, 35]. To identify TFs that regulate terpene biosynthesis we used a transient assay based on the transactivation of tomato terpene synthase promoters *in planta*.

## Results

### Assembly of RNAseq data and Genome Analyzer II transcript profiling

We created a tomato trichome EST database by sequencing a mixture of glandular and non-glandular trichome RNAs, derived from stems of *Solanum lycopersicum* cv. Moneymaker plants. The resulting cDNA was normalized prior to being used as input for 454 GS FLX Titanium pyrosequencing. A full plate was sequenced consisting of two halves: one with cDNAs originating from control plants and the other half with cDNAs originating from plants treated with JA. In total we obtained 979,076 high-quality reads with an average length of 337 bp. The reads from control and JA-treated samples were assembled *de novo* resulting in 27,195 contigs with an average length of 931 bp, leaving 24,187 reads unmatched (singletons), with an average length of 241 bp. Nucleotide sequences of the contigs were blasted against the Solanaceae Genomics Network (SGN) tomato database for annotation, using a local E-Blast tool; 3,295 contigs were not annotated.

For creating the transcript profiling databases with Genome Analyzer II, the same RNA material as for the 454 sequencing was used, but this time the cDNA derived from control and JA-treated stem trichomes was not normalized before being processed. We specifically obtained 5,631,975 3’ sequences from the Control sample and 5,882,547 from the JA-treated sample. 4,840,738 and 5,169,891 reads from the Control and JA-samples, respectively, were mapped to one unique contig of the trichome database. In addition, 38,699 (C) and 45,375 (JA) reads were mapped to multiple contigs and 791,237 (C) and 712,656 (JA) remained unmapped.

Both the 454 GS FLX Titanium reads and the Genome Analyzer II reads can be found in the Sequence Read Archive of NCBI (http://www.ncbi.nlm.nih.gov/sra) under accession number SRP041373.

### Annotation, gene ontology and protein families

In order to characterize the *S. lycopersicum* stem trichome transcriptome the unique contigs (27,195 ESTs) were submitted to homology searches (BLASTX) in the National Center for Biotechnology Information (NCBI) non-redundant protein database using Blast2GO [36]. 4,733 contigs did not return a BLASTX hit. The majority of the top hits were to protein sequences of *Vitis vinifera*, followed by *Populus trichocarpa*, *Ricinus communis* and *Solanum lycopersicum*.

Next, gene ontology (GO) and enzyme classifications (EC) were performed in order to classify the ESTs. It must be noted that one sequence could be assigned to more than one GO term. For the cellular component class the assignments were mostly given to cell and organelle (54,82% and 29,35% respectively; Additional file 1: Figure S1a). The highest percentage of molecular function GO terms were in binding and catalytic activity (42,96% and 41,38% respectively; Additional file 1: Figure S1c). In the biological processes, the majority of the GO terms was grouped into two categories- those of metabolic and cellular process (36,55% and 32,79% respectively; Additional file 1: Figure S1b). Finally, within the predicted ECs, the prevailing categories of enzymes were transferases and oxidoreductases (31,38% and 29,65% respectively; Additional file 1: Figure S1d).

The search of additional databases for protein families, domains, regions and sites was performed from Blast2GO via the InterPro EBI web server. The 30 top InterPro entries obtained are presented in Table 1. The most dominant class of enzymes was protein kinases. Abundantly represented were also cytochrome P450s.

**Table 1:** **Summary of the most common InterPro entries found in the** ***S. lycopersicum*** **stem trichome transcriptome**

InterPro	Frequency	Description
IPR011009	571	Protein kinase-like domain
IPR000719	521	Protein kinase, catalytic domain
IPR002290	336	Serine/threonine-/dual-specificity protein kinase, catalytic domain
IPR008271	286	Serine/threonine-protein kinase, active site
IPR020635	283	Tyrosine-protein kinase, catalytic domain
IPR016040	263	NAD(P)-binding domain
IPR013083	239	Zinc finger, RING/FYVE/PHD-type
IPR017441	187	Protein kinase, ATP binding site
IPR002885	181	Pentatricopeptide repeat
IPR015943	175	WD40/YVTN repeat-like-containing domain
IPR001841	172	Zinc finger, RING-type
IPR012677	172	Nucleotide-binding, alpha-beta plait
IPR016024	166	Armadillo-type fold
IPR001245	164	Serine-threonine/tyrosine-protein kinase catalytic domain
IPR000504	158	RNA recognition motif domain
IPR001680	153	WD40 repeat
IPR011046	152	WD40 repeat-like-containing domain
IPR011990	147	Tetratricopeptide-like helical
IPR011989	145	Armadillo-like helical
IPR001128	141	Cytochrome P450
IPR017986	133	WD40-repeat-containing domain
IPR017853	130	Glycoside hydrolase, superfamily
IPR012287	119	Homeodomain-related
IPR001611	115	Leucine-rich repeat
IPR009057	115	Homeodomain-like
IPR016196	112	Major facilitator superfamily domain, general substrate transporter
IPR012336	108	Thioredoxin-like fold
IPR013781	107	Glycoside hydrolase, subgroup, catalytic domain
IPR002213	102	UDP-glucuronosyl/UDP-glucosyltransferase
IPR002401	95	Cytochrome P450, E-class, group I

Finally, within Blast2GO, the EC numbers were classified in KEGG pathways, enabling the presentation of enzymatic functions in the context of the metabolic pathways in which they are part of (Blast2GO Tutorial, [37]). Among the pathways identified, the ones related to secondary metabolism are shown in Table 2. Lipid transfer proteins represented 0.19% of the tomato stem trichome transcripts.

**Table 2:** **KEGG pathways related to biosynthesis of secondary metabolites found in the** ***S. lycopersicum*** **stem trichome transcriptome**

KEGG pathway	EC nr	Enzyme name	Nr of sequences
*Terpenoid biosynthesis*	ec:1.1.1.208	(+)-neomenthol dehydrogenase	1
	ec:4.1.1.33	diphosphomevalonate decarboxylase	3
	ec:2.2.1.7	1-deoxy-D-xylulose-5-phosphate synthase	2
	ec:1.17.1.2	4-hydroxy-3-methylbut-2-enyl diphosphate reductase	1
	ec:2.7.7.60	2-C-methyl-D-erythritol 4-phosphate cytidylyltransferase	1
	ec:2.5.1.1	dimethylallyltranstransferase	1
	ec:1.17.7.1	(E)-4-hydroxy-3-methylbut-2-enyl-diphosphate synthase	1
	ec:2.7.1.148	4-(cytidine 5′-diphospho)-2-C-methyl-D-erythritol kinase	1
	ec:1.1.1.267	1-deoxy-D-xylulose-5-phosphate reductoisomerase	1
	ec:1.1.1.34	hydroxymethylglutaryl-CoA reductase (NADPH)	6
	ec:2.5.1.31	ditrans,polycis-undecaprenyl-diphosphate synthase	4
	ec:2.3.3.10	hydroxymethylglutaryl-CoA synthase	6
	ec:5.3.3.2	isopentenyl-diphosphate Delta-isomerase	2
	ec:2.5.1.32	phytoene synthase	2
*Phenylpropanoid biosynthesis*	ec:2.1.1.104	caffeoyl-CoA O-methyltransferase	4
	ec:1.11.1.7	peroxidases	56
	ec:3.2.1.21	beta-glucosidase	7
	ec:2.1.1.68	caffeate O-methyltransferase	1
	ec:1.14.13.11	trans-cinnamate 4-monooxygenase	1
	ec:6.2.1.12	4-coumarate---CoA ligase	4
*Flavonoid biosynthesis*	ec:2.1.1.104	caffeoyl-CoA O-methyltransferase	4
	ec:2.3.1.74	naringenin-chalcone synthase	2
	ec:1.14.11.23	flavonol synthase	7
	ec:1.14.13.88	flavonoid 3′,5′-hydroxylase	3
	ec:1.14.11.9	flavanone 3-dioxygenase	1
	ec:1.1.1.219	dihydrokaempferol 4-reductase	2
	ec:1.14.13.21	flavonoid 3′-monooxygenase	3
	ec:1.14.13.11	trans-cinnamate 4-monooxygenase	1
	ec:5.5.1.6	chalcone isomerase	1
*Alkaloid biosynthesis*	ec:4.3.3.2	strictosidine synthase	2
	ec:4.1.1.28	aromatic-L-amino-acid decarboxylase	4
	ec:1.14.11.20	deacetoxyvindoline 4-hydroxylase	1
	ec:2.6.1.42	branched-chain-amino-acid transaminase	3
*Steroid biosynthesis*	ec:1.14.21.6	lathosterol oxidase	1
	ec:2.1.1.41	sterol 24-C-methyltransferase	1
	ec:2.5.1.21	squalene synthase	2
	ec:5.3.3.5	cholestenol Delta-isomerase	1
	ec:2.1.1.6	catechol O-methyltransferase	1
	ec:1.1.1.145	3beta-hydroxy-Delta5-steroid dehydrogenase	2
	ec:1.14.14.1	unspecific monooxygenase	1
	ec:1.3.99.5	3-oxo-5alpha-steroid 4-dehydrogenase	4

Transcripts of enzymes involved in jasmonic acid biosynthesis and signaling pathways were also identified in the trichome database. Data for a selection of enzymes are presented in Table 3, including known JA marker genes such as *LOXA* (U09026), *AOC* (AW624058; [38]), *JAZ1* (EF591123; [39]) and *JAZ3* (EU194561; [40]).

**Table 3:** **List of selected enzymes involved in the jasmonic acid biosynthesis and signaling**

Abbreviation	Contig Nr	SGN Nr	Annotation	Transcript length (bases)	Expression values JA^*^	Expression values C^*^	Fold JA induction
LOXA^a^	6402	SGN-U592535	Lipoxygenase A	2837	35.2	1.6	22
AOC^a^	24817	SGN-U562649	Allene oxide cyclase	1645	1898.8	417.6	4.5
JAZ1^b^	6863	SGN-U579837	Jasmonate ZIM-domain 1	1156	12.2	0.7	17.4
JAZ3^c^	20751	SGN-U564446	Jasmonate ZIM-domain 3	986	18.87	2.3	8.2
COI1^a^	24353	SGN-U568988	Coronatine-insensitive 1	2260	11	8.5	1.29

^a^[38]: LOXA (U09026), AOC (AW624058), COI1 (NM_001247535), ^b^[39]: JAZ1 (EF591123), ^c^[40]: JAZ3 (EU194561).

*Expression values are in RPKM (reads per kilobase of transcript per million mapped reads).

**Figure 1:**
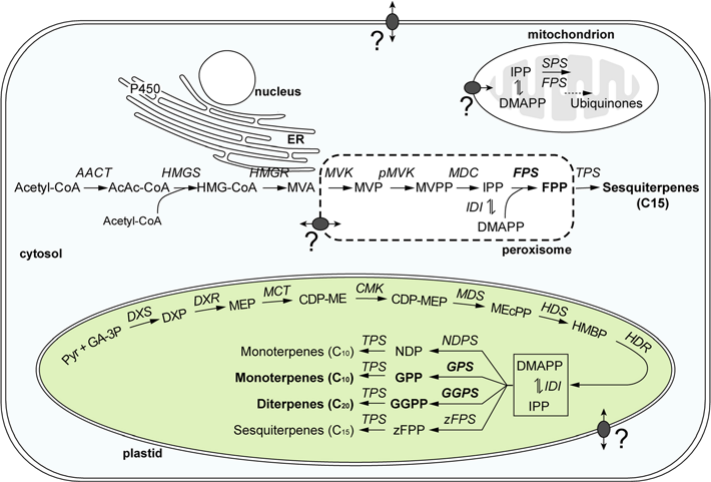
***Enzymes involved in the precursor biosynthesis for mono-(C10), sesqui-(C15) and di-(C20) terpenes*** . An explanation of the abbreviations used in the pathways and the GAII reads for each enzyme are shown in Tables 4 and 5, respectively. *ER*; endoplasmic reticulum, *TPS*; terpene synthase, *SPS*; solanesyl diphosphate synthase. The grey oval circles between organelles and at the cell membrane represent putative transporter systems. Peroxisomal localization of precursor enzymes of the MVA pathway has been previously reported in *Arabidopsis thaliana* and *Catharanthus roseus*[42–44].

A closer look was taken at the terpene biosynthesis pathway (Figure 1) in order to see if the precursor pathways were up-regulated by JA. As shown in Table 4, expression of some precursor genes in tomato was induced by JA although not strongly (max induction ~2.5-fold for *HDS*). As in other plants [41], genes encoding enzymes of the precursor pathways can belong to small gene families and it appears that expression levels and JA-inducibility of these members can vary. Transcript abundance of precursor genes is presented in Table 4 for comparison with the expression levels of the 13 terpene synthases (TPSs) found in stem trichomes Table 5).

**Table 4:** **Enzymes involved in the biosynthesis of precursors of mono-, sesqui- and diterpenes**

Abbreviation	Name	Chr	SGN nr	Expression values JA^*^	Expression values C^*^	Fold JA induction
AACT	acetoacetyl-coenzyme A thiolase	5	SGN-U566720	351.04	275.21	1.27
		7	SGN-U566719	126.3	133.52	0.94
HMGS	3-hydroxy-3-methylglutaryl-CoA synthase	8	SGN-U579858	14.96	15.98	0.93
		8	SGN-U578388	205.34	123.57	1.66
HMGR	3-hydroxy-3-methylglutaryl-CoA reductase	2	SGN-U580675	2.2	1.06	2.07
		2	SGN-U578017	19.2	22.19	0.86
		3	SGN-U579319	43.35	24.5	1.77
MVK	mevalonate kinase	1	SGN-U567385	198.89	125	1.59
pMVK	phosphomevalonate kinase	8	SGN-U583971	46.92	54.51	0.86
MDC	mevalonate diphosphate decarboxylase	4	SGN-U587221	3.4	3.19	1.06
		11	SGN-U581971	26.68	35.51	0.75
DXS1	1-deoxy-D-xylulose 5-phosphate synthase 1	1	SGN-U567647	24.14	35.33	0.68
DXS2	1-deoxy-D-xylulose 5-phosphate synthase 2	11	SGN-U582996	37.23	22.72	1.64
DXR	1-deoxy-D-xylulose 5-phosphate reductoisomerase	3	SGN-U585813	657.7	497.34	1.32
MCT	4-diphosphocytidyl-2-C-methyl-D-erythritol synthase	1	SGN-U566797	107.95	121.45	0.89
CMK	4-diphosphocytidyl-2-C-methyl-D-erythritol kinase	1	SGN-U583224	275.9	237.57	1.16
MDS	2C-methyl-D-erythritol 2,4-cyclodiphosphate synthase	8	SGN-U568497	36.72	41.73	0.88
HDS	1-hydroxy-2-methyl-2-(E)-butenyl 4-diphosphate synthase	11	SGN-U567167	30.43	12.25	2.48
HDR	1-hydroxy-2-methyl-2-(E)-butenyl 4-diphosphate reductase	1	SGN-U580658	2916.93	2720.54	1.07
IDI	isopentenyl diphosphate isomerase	5	SGN-U569721	3.4	1.95	1.74
		4	SGN-U577516	957.4	749.11	1.28
GPS	geranyl diphosphate synthase	8	SGN-U573523	2.71	3.36	0.8
NDPS (CPT1)	neryl diphosphate synthase	8	SGN-U583641	2810.51	4696.4	0.6
FPS	farnesyl diphosphate synthase	12	SGN-U580757	11.05	9.76	1.13
		10	SGN-U578686	0.68	1.24	0.55
		10	SGN-U581576	15.98	25.21	0.63
GGPS	geranyl geranyl diphosphate synthase	4	SGN-U571085	6.12	7.46	0.82
		9	SGN-U575882	63.58	66.05	0.96
		2	SGN-U573348	17.17	34.8	0.49
CPT3	*cis*-prenyl transferase 3	3	SGN-U572901	14.45	17.58	0.82
CPT4	*cis*-prenyl transferase 4	10	SGN-U568982	9.52	7.28	1.3
CPT5	*cis*-prenyl transferase 5	10	SGN-U585528	120.17	70.3	1.7
CPT7	*cis*-prenyl transferase 7	6	SGN-U574892	291.53	471.59	0.62

*Chr*; chromosome.

*Expression values are in RPKM (reads per kilobase of transcript per million mapped reads).

For an overview of the biosynthetic pathway see Figure 1.

**Table 5:** **Terpene synthases (TPS) found in** ***S. lycopersicum*** **stem trichomes**

TPS	Transcript length (bases)	Expression values JA^*^	Expression values C^*^	Fold JA induction
3	2099	15.8	2.3	6.87
5	2186	103.18	46.7	2.2
7	1069	0.5	0.18	2.78
9	2011	3060.75	2359.03	1.3
12	407	4.08	2.66	1.53
16	1868	18.36	16.87	1.09
17	1190	4.25	3.55	1.2
19	776	44.7	37.46	1.19
20	1148	142.79	106.89	1.33
24	854	0.17	0.18	0.94
31	1991	0.68	0.18	3.78
39	1131	12.07	5.68	2.12
41	2368	71.57	66.58	1.07

*Expression values are in RPKM (reads per kilobase of transcript per million mapped reads).

For *SlTPS8* no transcripts were identified in the stem trichome database although by Q-RT-PCR minimal expression has been observed [45].

### Selection of transcription factors potentially involved in regulating terpene synthases

Based on the annotated contigs 743 transcription factors of different classes were found in the trichome database: 69 WRKY, 151 MYB, 8 MYC, 52 bZIP, 9 ARF, 71 ERF, 17 ZnF, 28 bHLH, 12 MADS, 1 NAC and 325 of unknown function/class. Out of those, 151 were up-regulated (>1.5x) by the treatment with JA, 119 were down-regulated (<0.67x) and expression of 473 TFs remained unaltered. Since JA is known to play a role in the plant’s direct and indirect defenses we were interested in those transcription factors that were induced by JA and could therefore potentially be involved in up-regulating terpene biosynthesis. 56 of the TFs that were up-regulated by JA showed an induction higher than 2-fold. The sequence of these 56 TFs was blasted against the tomato genomic sequence (Solanaceae Genomics Network, SGN) and complete ORFs were constructed when possible (GENSCAN, [46]), if not provided by the RNAseq. These sequences were submitted to homology search after translation against the NCBI database for identifying conserved domains. From this analysis 16 TFs (Table 6) were selected for further investigation as follows: we focused on classes of TFs involved in the regulation of terpenoids identified so far in other plant species- namely TFs of the APETALA2 class [25, 30, 31], WRKY class [27–29] and MYC class [26, 33]. In total eleven transcription factors of the AP2 class, four of the WRKY class and one of the MYC class, although it only showed a 1.4-fold induction, were selected for further investigation of their potential involvement in regulating expression of terpene synthases.

**Table 6:** **List of selected** ***S. lycopersicum*** **transcription factors (TF) potentially involved in terpene biosynthesis**

Name	Contig nr	SGN nr	Annotation	Transcript length (bases)	Expression values JA^*^	Expression values C^*^	Fold JA induction
SlAP2_9	83	SGN-U572361	ERF (ethylene response factor) subfamily B-3 of ERF/AP2 TF family	371	9.69	4.08	2.37
SlAP2_2	1719	SGN-U596590	DREB subfamily A-1 of ERF/AP2 TF family	422	13.6	1.95	6.97
SlAP2_6	5289	SGN-U563871	AP2 domain-containing TF	933	45.39	12.78	3.55
SlAP2_3	7031	SGN-U563215	DREB subfamily A-1 of ERF/AP2 TF family	981	5.95	1.42	4.19
SlAP2_7	7865	SGN-U587768	DREB subfamily A-4 of ERF/AP2 TF family	775	2.2	0.71	3.09
SlAP2_4	10714	SGN-U586437	ERF (ethylene response factor) subfamily B-3 of ERF/AP2 TF family	611	2.72	0.71	3.83
SlAP2_10	14672	SGN-U585539	AP2 domain-containing TF	297	18.87	7.99	2.36
SlAP2_5	16204	SGN-U577088	ERF (ethylene response factor) subfamily B-4 of ERF/AP2 TF family	523	0.68	0.18	3.78
SlAP2_11	25582	SGN-U584756	ERF (ethylene response factor) subfamily B-2 of ERF/AP2 TF	660	1.19	0.53	2.24
SlAP2_1	25985	SGN-U586438	ERF (ethylene response factor) subfamily B-3 of ERF/AP2 TF family	942	16.32	0.53	30.8
SlAP2_8	26482	SGN-U581852	Ethylene-responsive element-binding factor 4 homolog	788	58.81	24.32	2.42
SlWRKY22	9827	SGN-U565154	WRKY family TF	1366	0.68	0.18	3.78
SlWRKY28	10561	SGN-U584367	WRKY family TF	1326	0.5	0.18	2.78
SlWRKY78	13200	SGN-U565157	WRKY family TF	1099	1.19	0.18	6.61
SlWRKY73	20918	SGN-U571278	WRKY family TF	1453	0.5	0.18	2.78
SlMYC1	24332	SGN-U576396	MYC TF	2174	32.47	22.7	1.43

*Expression values are in RPKM (reads per kilobase of transcript per million mapped reads).

### Tissue specificity and JA responsiveness of selected transcription factors

**Figure 2:**
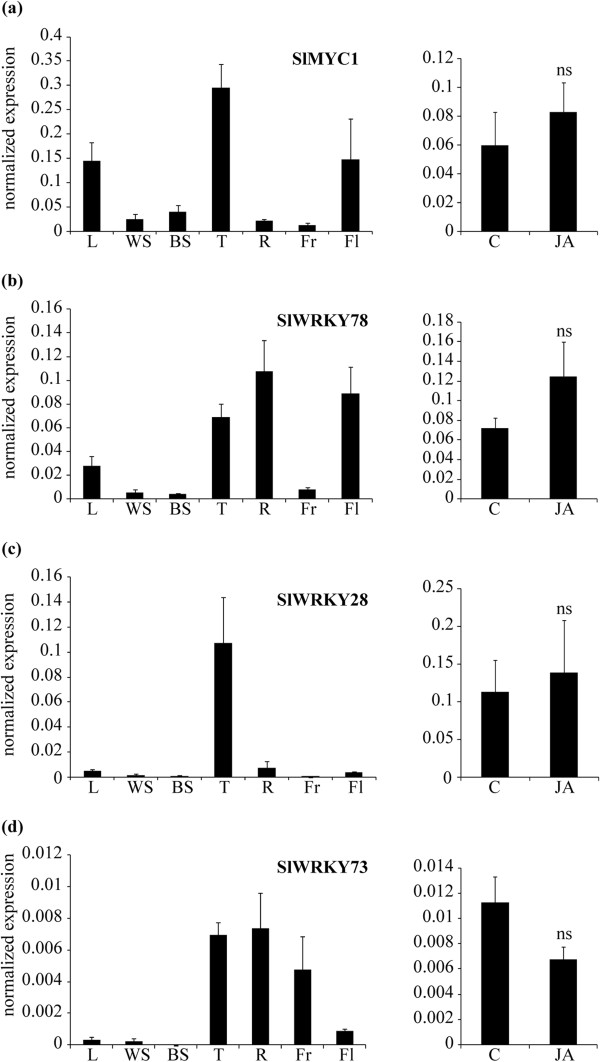
***Tissue specific expression and JA induction of selected TFs.*** Transcript levels for **(a)** *SlMYC1* **(b)** *SlWRKY78*, **(c)** *SlWRKY28* and **(d)** *SlWRKY73* as determined by Q-RT-PCR. Mean values (+SE) of 3 biological replicas are shown, normalized for *Actin* expression. *L*; leaf, *WS*; whole stem, *BS*; bald stem, *T*; stem trichomes, *R*; root, *Fr*; fruit, *Fl*; flower; *C*; control and *JA*; jasmonic acid induced stem trichomes. *ns*; not significant according to *T*-test.

The sixteen candidate TFs should ideally be trichome-specifically expressed and possibly induced by jasmonic acid. In order to investigate the expression pattern of these genes, cDNA was synthesized from different *S. lycopersicum* cv. Moneymaker organs and tissues: leaves, stems, isolated stem trichomes and roots from 4-week-old plants, as well as flowers and fruit of mature plants. In Figure 2 transcript levels, as determined by Q-RT-PCR, are presented for four of the sixteen selected transcription factors. For the other twelve candidate TFs expression in the trichomes was much lower than that in the other organs/tissues and these were excluded from further analysis. TF *SlMYC1* (KF430611) was predominately expressed in trichomes, but also in leaves and flowers (Figure 2a). *SlWRKY78* was expressed in leaves, trichomes, roots and flowers (Figure 2b). *SlWRKY28* was a trichome-specific gene (Figure 2c) and *SlWRKY73* was expressed in trichomes, roots and fruit (Figure 2d). Q-RT-PCR analyses indicated that none of the selected transcription factors was significantly induced by JA according (Figure 2). *SlWRKY73* expression appeared to be approximately 1.7-fold reduced in JA treated plants (p = 0.07).

### SlMYC1 and SlWRKY73 can transactivate terpene synthase promoters in *Nicotiana benthamiana* leaves

**Figure 3:**
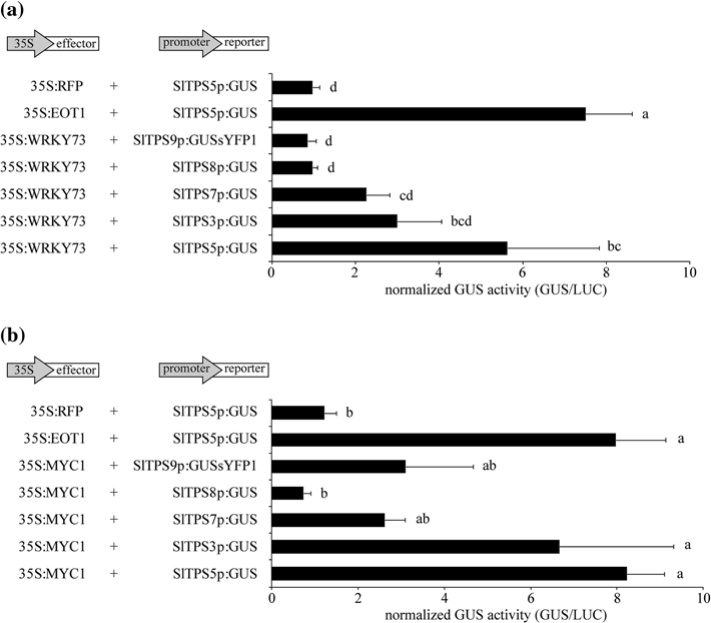
***Transactivation of terpene synthase promoters by SlMYC1 and SlWRKY73 in*** **N. benthamiana** ***leaves*** . Normalized GUS activity after co-infiltration with *A. tumefaciens* harboring the **(a)** 35S:WRKY73 or **(b)** 35S:MYC1 effector construct and various promoter:GUS reporter constructs. The 35S:SlEOT1 and 35S:RFP effector constructs were used as positive and negative control, respectively. The bars represent the obtained mean values and the error bars the standard error (n = 3). *RFP*; red fluorescent protein. Letters indicate significant differences (ANOVA, *P* < 0.05 according to Tuckey’s B posthoc test). Representative results from three experiments are shown.

In order to investigate whether these TFs could activate a selection of terpene synthase promoters, a transient assay in *Nicotiana benthamiana* leaves was used, which has been previously shown to work for the interaction between the zinc finger-like transcription factor Expression of Terpenoids 1 (SlEOT1) and the *SlTPS5* promoter [47]. In the reporter construct, expression of β-glucuronidase (*uidA*, GUS) is driven by the glandular trichome-specific promoter of *SlTPS5*. Co-infiltration with the 35S:SlEOT1 effector construct resulted in transactivation of the *SlTPS5* promoter, leading to GUS expression in this heterologous system (Figure 3). As negative control for the effector, a 35S:RFP construct was used. Various other reporter constructs with promoters of other terpene synthases- *SlTPS3*, *SlTPS7* and *SlTPS8*- driving expression of GUS or a GUSsYFP1 fusion (*SlTPS9*) were included in the analyses.

As shown in Figure 3a, SlWRKY73 could transactivate the *SlTPS5* promoter, albeit to a lower extent than SlEOT1. SlWRKY73 transactivated the *SlTPS3* and *SlTPS7* promoters only weakly, and the *SlTPS8* and *SlTPS9* promoters not at all (35S:RFP negative controls shown in Additional file 1: Figure S2). SlWRKY78 or SlWRKY28 did not transactivate any of the terpene synthase promoters (Additional file 1: Figure S3).

SlMYC1 could transactivate all terpene synthase promoters tested except *SlTPS8*. Transactivation of the trichome-specific *SlTPS5* and *SlTPS3* promoters was strongest (Figure 3b; 35S:RFP negative control shown in Additional file 1: Figure S2). However, it should be noted that GUS activity of a promoter driving the GUSsYFP1 fusion was lower than when the same promoter driving GUS alone was transactivated by an effector construct (data not shown), possibly because the fusion protein was less stable or produced. Therefore, transactivation by SlMYC1 of the trichome-specific *SlTPS9* promoter was potentially stronger than that detected here.

### SlMYC1 and SlEOT1 act synergistically on the *SlTPS5* promoter in *N. benthamiana* leaves

**Figure 4:**
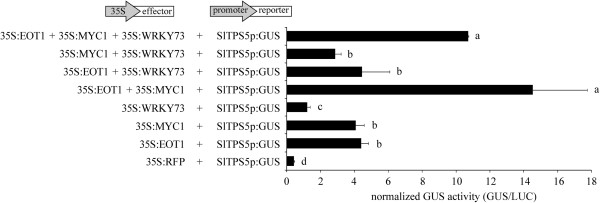
***Transactivation of SlTPS5 promoter by SlEOT1, SlMYC1, SlWRKY73 or combination thereof in*** **N. benthamiana** ***leaves*** . Normalized GUS activity after co-infiltration with *A. tumefaciens* harboring the 35S:EOT1, 35S:MYC1, 35S:WRKY73 effector constructs or combination thereof and the SlTPS5p:GUS reporter construct. The 35S:RFP effector construct was used as negative control. The bars represent the obtained mean values and the error bars the standard error (n = 4). *RFP*; red fluorescent protein. Letters indicate significant differences (ANOVA, *P* < 0.05 according to Tuckey’s B posthoc test). Representative results from two experiments are shown.

Since SlEOT1, SlMYC1 and SlWRKY73 were shown in separate experiments to be able to transactivate the *SlTPS5* promoter (Figure 3), we investigated what effect a combination of these transcription factors would have on the transactivation of this promoter. To this end, *Agrobacterium* cultures carrying the CaMV 35S-driven effector constructs were mixed in pairs or all three together and combined with the SlTPS5p: GUS reporter construct and infiltrated in *N. benthamiana* leaves (Figure 4). Interestingly, co-expression of SlEOT1 and SlMYC1 almost tripled the transactivation of *SlTPS5* promoter compared to the effect of each TF alone. Adding SlWRKY73 did not have an additional effect, but rather seemed to have a negative effect on the combinatorial action of the other two TFs, although not at a statistically significant level (Figure 4).

## Discussion and conclusions

RNA sequencing of *S. lycopersicum* stem trichomes led to the identification of one MYC bHLH and one WRKY transcription factor that can transactivate several terpene synthase promoters. The observation that SlMYC1 acts synergistically with SlEOT1 in the transactivation of the *SlTPS5* promoter suggests a complex regulatory network for terpene biosynthesis.

### High-throughput sequencing of *Solanum lycopersicum* stem trichomes

We used massive parallel pyrosequencing on the 454 GS FLX Titanium platform to sequence *S. lycopersicum* stem trichome RNAs with the goal to identify transcription factors involved in terpene biosynthesis. We used normalized cDNA to maximize representation of low abundant transcripts and reduce representation of highly abundant transcripts. Attempts to map the obtained reads to the publicly available mixed tissue SGN database led to a high percentage of unmapped reads and assignment of the same reads to multiple unigenes and therefore the reads were assembled *de novo*. 2.5% of the reads could not be matched and were not used in further analysis. 87.9% of the resulting contigs were subsequently annotated after blasting against the SGN tomato database using a local E-Blast tool. In this database we identified annotated enzymes involved in several metabolic pathways (Additional file 1: Table S1). In short, compared to the study published by McDowell and colleagues [3] on *S. lycopersicum* cv. M82 trichomes, we identified in Moneymaker trichomes cDNAs encoding enzymes involved in for example the TCA cycle, starch and sucrose metabolism (Additional file 1: Table S1), as well as secondary metabolite biosynthesis (Table 2). Photosynthesis related genes were also identified but were not as prevalent (Additional file 1: Table S1) as in M82 trichomes. Such differences could originate from the fact that in our study we used a mix of Moneymaker trichome types, including stalks, whereas McDowell and colleagues focused on comparing different types of trichomes between *Solanum* species and so clipped off and analyzed only the secretory cells of glandular trichomes [3].

Furthermore we created an expression profiling database using Illumina sequencing in order to obtain genes regulated by JA. The success of the JA treatment is evident by the high induction of known JA markers, some of which are presented in Table 3 (LOXA, AOC [38]; JAZ1 [39]; JAZ3 [40]).

### Jasmonic acid regulation of the terpene biosynthesis pathway in tomato trichomes

In order to investigate whether in stem trichomes of tomato Moneymaker plants, regulation of terpene biosynthesis by JA is also on the precursor level besides on the level of individual TPSs [21], the quantitative database was mined for enzymes of the precursor pathways. The copy number of these genes varies between different plant species [41] and, as shown in Table 4, different family members can vary in their expression levels and/or JA-inducibility. For example 1-deoxy-d-xylulose 5-phosphate synthase (DXS), in contrast with *Arabidopsis*, which contains a single functional gene, has diversified into two isogenes in other plant species including tomato [48]. Whereas *SlDXS1* is ubiquitously expressed, *SlDXS2* is expressed only in a few tissues and in leaf trichomes its transcript abundance is much higher than that of *SlDXS1*[49], although this is not the case in stem trichomes (Table 4). Furthermore, *SlDXS2* is moderately induced by wounding in the cultivar Moneymaker [49], which correlates with the observed moderate induction of *SlDXS2* by JA (~1.6-fold, Table 4). *SlDXS2* expression is also approximately threefold upregulated in the tomato cultivar Castlemart upon feeding by *Manduca sexta* larvae [50].

The regulation of precursor genes of the MEP pathway by wounding, hormones or elicitors has been demonstrated in various plant species [49–54]. Similarly, evidence for the regulation of precursor biosynthesis of the mevalonate (MVA) pathway is also abundant [55–60]. For example, HMGR enzyme activity and protein level were shown to increase by fungal infection in potato tubers and sweet potato root [59]. Furthermore, *HMGR1* expression was induced by treatment with MeJA in potato, whereas *HMGR2* expression was reduced [56]. In response to caterpillar herbivory, transcripts of *HMGR1* were reduced in alfalfa [60]. Our results show that in tomato stem trichomes *HMGR1* and *HMGR3* were induced by JA treatment approximately 2-fold, whereas expression of *HMGR2* remained unaltered (Table 4). None of the prenyl diphosphate synthases were induced in tomato trichomes by JA treatment, whereas two seemed to be downregulated (*FPS*, SGN-U578686; and *GGPS*, SGN-U573348; Table 4). We did not find any transcripts for *GGPS1* (SGN-U574849) in our stem trichome database, although it has been shown to be induced in tomato leaves by JA-treatment [61]. Finally, from the very recently identified *cis*-prenyltransferases only *CPT5*, that produces medium-length chain polyisoprenoids [62], was upregulated by JA, 1.7-fold (Table 4).

### Identification of transcription factors involved in regulating terpene synthases in tomato trichomes

Our primary aim was to identify transcription factor(s) that regulate terpene biosynthesis. Based on the annotated contigs, 2.7% of the transcripts in the tomato stem trichomes encode transcription factors. For comparison, in *Arabidopsis thaliana* ~6% of the genes in all tissues encode TFs (TAIR10 genome release, [63]). Since JA is essential for establishing indirect defense responses in tomato [34, 35] and the induction of terpene synthases in trichomes [16, 21], we hypothesized that TFs involved in the regulation of terpene biosynthesis would also be JA-inducible genes. Most of the transcription factors known to be involved in regulation of terpenoid pathways are jasmonate-inducible and of the APETALA2, WRKY or MYC class [25–30, 33]. However, in Arabidopsis it was recently shown that two MYC transcription factors (AtMYC3 and AtMYC4), which act additively with AtMYC2 in the activation of JA responses, are, in contrast to *AtMYC2*, only marginally induced by JA treatment [64]. Based on all the above, the initial selection of transcription factors to be analyzed from our quantitative stem trichome database was limited to TFs of the AP2, WRKY and MYC class that showed a 2-fold or higher induction by JA treatment (2.2-fold was the induction rate of control gene *SlMTS1*; [16], renamed *SlTPS5*[21]; Table 5). None of the MYC transcription factors of our database showed induction higher than 2, so for further analysis the closest homolog of AtMYC2 [32] was selected, as it has been shown to activate the *AtTPS11* and *AtTPS21* promoters [33]. After discarding TFs that were not trichome-specific or did not show highest expression in trichomes, the list was narrowed down to four candidate transcription factors. According to the Q-RT-PCR data however, none of these TFs was significantly induced by JA treatment (Figure 2). Since the numbers of sequence reads of these genes is very low both in the Control and JA samples (Table 6), the fold-induction in the Illumina experiments must have been overestimated.

### SlMYC1 and SlWRKY73 transactivate terpene synthase promoters in planta

**Figure 5:**
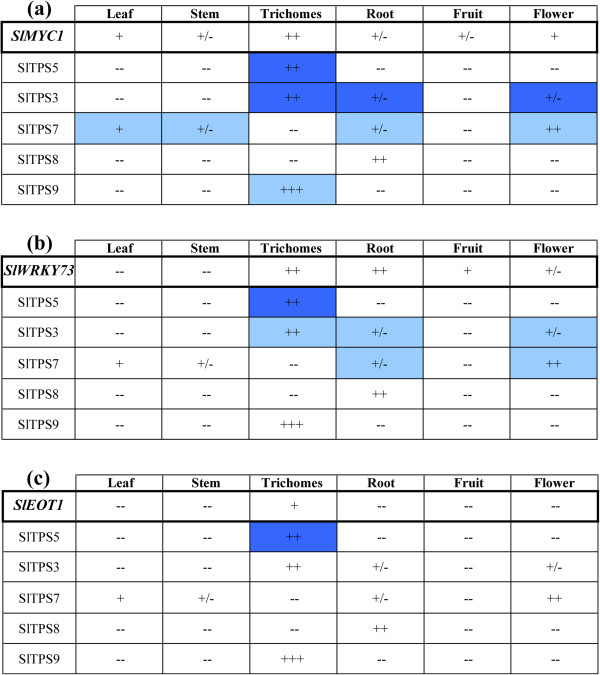
***Expression patterns and activation overview of TFs and TPSs.*** Putative positive interaction of the respective SlTPS promoters by transcription factors **(a)** SlMYC1, **(b)** SlWRKY73 and **(c)** SlEOT1 is indicated by a colored box that represents the tissue in which they are co-expressed. Expression in the various tissues is indicated by +++, ++, +, +/− and -- according to Q-RT-PCR values. Darker shaded boxes indicate a stronger transient activation of the TPS promoter by the TF in *N. benthamiana* leaves. *EOT1*; Expression of Terpenoids 1 [47].

A specific indication of whether any of these TFs are involved in regulating terpene biosynthesis would be the activation of terpene synthase promoters by the transcription factor. In transient activation assays in *N. benthamiana* leaves two of the four selected transcription factors were able to transactivate at least one terpene synthase promoter. SlWRKY73 showed strongest transactivation of the *SlTPS5* promoter and in lesser extent of the *SlTPS3* and *SlTPS7* promoters (Figure 3a). Although *SlWRKY73* is expressed highly in roots (Figure 2), SlWRKY73 could not transactivate the promoter of *SlTPS8* that is mainly expressed in roots. It could also not transactivate the trichome-specific sesquiterpene synthase *SlTPS9* promoter so it is possible that SlWRKY73 can transactivate only monoterpene synthases or at least not the sesquiterpene synthase tested here (Figure 3a). As shown in Figure 5*SlWRKY73* and the respective TPSs that it can transactivate are co-expressed in various tissues where the regulation could take place in the plant.

SlMYC1 showed strongest transactivation of *SlTPS5* and *SlTPS3* and to a lesser extent of *SlTPS7* and *SlTPS9* but no transactivation of *SlTPS8* promoter (Figure 3b), although *SlMYC1* is also expressed in the root, albeit not strongly (Figure 2). As shown in Figure 5*SlMYC1* is expressed (at different levels) in every plant tissue and SlMYC1 is able to activate all the terpene synthase promoters tested except one, so it seems to be a regulator of multiple TPSs, in contrast to *SlEOT1* that is only expressed in the glandular trichomes and can specifically transactivate the *SlTPS5* promoter and none of the other TPS promoters tested (Figure 5, [47]). The other two selected TFs (SlWRKY78 and SlWRKY28; Additional file 1: Figure S3) were not able to significantly transactivate any of the tested terpene synthase promoters. However it cannot be excluded that these TFs were not expressed in the transient assay.

One question that arises is, of course, where SlWRKY73 and SlMYC1 bind on these terpene synthase promoters. In the promoter sequence of *SlTPS5*, *SlTPS3* and *SlTPS7*[47] there are five, four and one W-boxes (TGAC(C/T)) respectively (PLACE; [65], Additional file 1: Table S2), which could serve as potential binding site(s) for SlWRKY73. Furthermore, *SlTPS5* promoter contains two G-box-like elements (CAC**A**TG instead of the canonical CACGTG), one T/G-box element (AACGTG) and one T/G-box-like element (**T**ACGTG) (Additional file 1: Table S2), which could potentially be the binding site(s) of SlMYC1. The promoter of *SlTPS3*, with which SlMYC1 interacts less strongly, contains one G-box-like element and one T/G-box element (Additional file 1: Table S2). The *SlTPS7* promoter, which SlMYC can also activate, contains one T/G-box (Additional file 1: Table S2). The *SlTPS9* promoter [47] however, does not contain any of these elements, which could indicate the existence of an uncharacterized motif to which SlMYC1 binds. When using the motif search program MEME [66] with all four promoters that SlMYC1 can activate, one 8 bp motif was identified in the plus or minus (for *SlTPS9*) orientation: CTAGG(T/A)(A/G)G. The validation of a (putative) regulatory element as the binding site for these TFs would require extensive further experimentation. However, since our transactivation assays do not indicate direct binding, the TF-TPS promoter interactions observed in the ATTAs, could take place through an additional protein. To address the issue of which terpene synthases (and possibly other genes as well) these TFs regulate, we are currently starting the more laborious but more conclusive approach of creating stably transformed silenced and overexpressing plants.

### SlMYC1 acts synergistically with SlEOT1 in the transactivation of the *SlTPS5* promoter

Interestingly, SlEOT1 and SlMYC1 acted synergistically in the transactivation of the *SlTPS5* promoter (Figure 4). Combinatorial control of transcriptional regulation is commonly found in plants and other eukaryotes [67]. For example, in abscisic acid (ABA) signaling, the 67 bp promoter region of the dehydration-responsive gene *rd22* contains a MYC and a MYB recognition site, where AtMYC2 and AtMYB2 can bind, respectively. In *Arabidopsis* leaf protoplasts it was shown that these TFs could individually activate transcription of *β-glucuronidase* driven by this 67 bp promoter region of *rd22* and that the transient activation was stronger when AtMYC2 and AtMYB2 were combined [68]. Transgenic plants overexpressing these TFs each showed ABA hypersensitivity but the effect was more profound in plants overexpressing both TFs [69].

Given the fact that *SlMYC1* and *SlEOT1* are not induced by JA (Figure 2, [47]) and yet the proteins can transactivate the JA-inducible *SlTPS5* promoter indicates that they could be regulating the steady-state transcription of *SlTPS5*. These TFs might however also be involved in the enhanced *SlTPS5* expression by interacting with other, inducible TF(s). From the well-studied cases of transcriptional regulation in *Catharanthus roseus*[25–27] and Arabidopsis [32, 33, 64] it has becomes clear that it usually involves a network of TFs. In *Solanum lycopersicum* we are only just starting to unravel the complexity of transcriptional regulation of terpene biosynthesis.

## Methods

### Hormone treatment and RNA isolation

Tomato plants (*Solanum lycopersicum* cultivar Moneymaker) were grown in soil in a greenhouse with day/night temperatures of 23°C/18°C and a 16/8 h light/dark regime for four weeks. They were then sprayed either with JA solution (1 mM JA; Duchefa, NL, in tap water + 0,05% SilwetL-77; GE Silicones, VA, USA) or with control solution (0,05% SilwetL-77 in tap water). Stem pieces were collected 30 min, 2 h, 8 h and 24 h later for pyrosequencing or 24 h later for expression analyses and trichomes were isolated by shaking the stems in liquid nitrogen. Total RNA was isolated using TRIzol (Invitrogen, Paisley, UK) according to the manufacturer’s instructions. Equal amount of trichome RNA from the different time points was pooled creating the control (C) and JA samples. RNA used for pyrosequencing was then purified on a RNeasy Plant column (Qiagen, Valencia, CA, USA).

### Transcriptome database construction

RNA quality was determined with the Agilent RNA pico chip (Agilent Technologies, Waldbronn, Germany). Synthesis and amplification of cDNA was performed using the SMART PCR cDNA Synthesis and Advantage 2 PCR kits (Clontech Inc., CA, USA) according to the manufacturer’s instructions with some modifications of adapters to eliminate 3′ poly(A)-stretches prior to sequencing. cDNA quality was determined with the Agilent DNA 7500 chip (Agilent Technologies, Waldbronn, Germany) or on an 1% agarose/EtBr gel. Normalization of the cDNA was carried out using the Evrogen TRIMMER kit (Evrogen, Moscow, Russia) according to the manufacturer’s protocol. The normalization efficiency was determined both on an agarose/EtBr gel (1%) and with an Agilent DNA 7500 chip. The cDNA was purified and concentrated using the Qiaquick PCR purification kit (Qiagen, Valencia, CA, USA). cDNA shearing and FLX Titanium library preparation was carried out using the Roche GS FLX Titanium General Library Preparation Method kit (Roche Diagnostics, Mannheim, Germany) according to the manufacturer’s protocol. The size range of the fragments was determined with an Agilent DNA 1000 chip (Agilent Technologies, Waldbronn, Germany). Exclusion of smaller-sized fragments was performed using the double SPRI method as described in the Roche GS FLX Titanium General Library Preparation protocol (Roche Diagnostics, Mannheim, Germany). End-polishing, small fragment removal, library immobilization, fill-in reaction and single-stranded library isolation was performed using the GS FLX Titanium General Library Preparation Method kit (454 Life Sciences, Roche Diagnostics, Mannheim, Germany) according to manufacturer’s instructions.

### Expression profiling database construction

Starting from the same total RNA samples (C and JA, *see* above), mRNA was amplified and purified using the MessageAmp II aRNA Amplification kit (Applied Biosystems/Ambion, CA, USA) according to manufacturer’s instructions. RNA quality was determined with the Agilent RNA pico chip (Agilent Technologies, Waldbronn, Germany). Synthesis of cDNA was performed using the MessageAmp II aRNA Amplification kit (Applied Biosystems/Ambion, CA, USA) according to manufacturer’s instructions with modifications of the adapters to enable sequencing of 3′ cDNA ends. cDNA was purified with the Qiaquick PCR purification kit (Qiagen, Valencia, CA, USA). cDNA quality was determined with the Agilent DNA 7500 chip (Agilent Technologies, Waldbronn, Germany) or on an 1% agarose/EtBr gel. Shearing and ligation was carried out using standard Illumina PE adapters containing a specific sample ID tag. Adapter-ligated cDNA fragments were column purified with the Qiaquick PCR purification kit (Qiagen, Valencia, CA, USA). The size range of the fragments was determined with an Agilent DNA 1000 chip (Agilent Technologies, Waldbronn, Germany). Exclusion of smaller-sized fragments was performed using a single SPRI procedure as described in the Agencourt Ampure PCR Purification protocol (Agencourt Bioscience Corporation, MA, USA). The size range of single-stranded fragments was determined with an Agilent RNA pico 6000 chip (Agilent Technologies, Waldbronn, Germany). Expression profiling was performed using the Illumina Genome Analyzer II System (Illumina, USA).

### Databases assembly, EST annotation and homology searches

The 454 sequencing reads (Control and JA combined) were assembled into contigs *de novo* by Vertis Biotechnologie AG, Germany using the CLCbio software [70]. Nucleotide sequences of the contigs were then blasted against the SGN unigenes v2 tomato database (ftp.solgenomics.net/unigene_builds/combined_species_assemblies/tomato_species) for annotation, using a local Eblast tool (*E* value 1e–9). The GA II reads (Control and JA separately) were mapped to the annotated contigs of the 454 sequencing trichome database by Vertis Biotechnologie AG, Germany.

The resulting contigs were also imported in the bioinformatics tool Blast2GO v.2.5.0 [37] and were blasted against the National Center for Biotechnology Information (NCBI) non-redundant protein database BLASTX (*E* value 1e–3). Further analyses with this tool included functional annotation by Gene Ontology (GO) terms and Enzyme Commission numbers (EC code), InterPro terms (InterProScan; [71]) and metabolic pathways (Kyoto Encyclopedia of Genes and Genomes, KEGG; [72]).

### cDNA synthesis and quantitative-real time-PCR

DNA was removed from RNA with DNAse (Ambion, Huntingdon, UK) according to the manufacturer’s instuctions and cDNA was synthesized from 1.5 μg RNA using M-MuLV H^−^ Reverse Transcriptase (Fermentas, St. Leon-Rot, Germany). For Q-RT-PCR, cDNA equivalent to 100 ng total RNA was used as template in 20 μl volume and reactions were performed in the ABI 7500 Real-Time PCR System (Applied Biosystems) using the Platinum SYBR Green qPCR SuperMix-UDG kit (Invitrogen, Paisley, UK) with the following cycling program: 2 min 50°C, 7 min 95°C, 45 cycles of 15 sec at 95°C and 1 min at 60°C, followed by a melting curve analysis. Primer pairs were tested for amplification kinetics and linearity with a standard cDNA dilution curve and new primers were designed if necessary. Expression levels were normalized using *ACTIN* (SGN-U579547) mRNA levels. Effects of JA on gene expression were analyzed in three biological replicates by *T*-test using PASW Statistics 17.0 [73]. The homogeneity of variance was tested by Levene’s test.

### Cloning, construct design and ATTAs

TFs *SlMYC1* (KF430611; sequence of the full-length ORF obtained from the 454 trichome database), *SlWRKY28* and *SlWRKY73* ([74]; Additional file 1: Figure S4) were cloned between restriction sites *Nco*I (at the ATG) and *Sac*I (at the 3′ end of the sequence) in front of the *Nos* terminator in vector pKG1662 (KeyGene, Wageningen, NL; for a map of the vector see patent nr US2011/0113512A1) driven by the CaMV 35S promoter. TF *SlWRKY78* ([74]; Additional file 1: Figure S4) was cloned downstream of the CaMV 35S promoter in vector pJVII, a pMON999-based vector (Monsanto, St. Louis, MO) with a modified multiple cloning site (MCS), between restriction sites *Xba*I (at the ATG) and *Bsr*GI (at the 3′ end of the sequence). All constructs were verified by sequencing and then the expression cassettes containing 35S promoter, cDNA of interest and *nos* terminator were transferred to the MCS of the binary vector pBINplus [75] between *Hind*III and *Sma*I restriction sites. The final constructs were transformed to *Agrobacterium tumefaciens* GV3101 (pMP90). The promoter:GUS constructs used in the transient transactivation assay have been described elsewhere [47]. The *A. tumefaciens* transient transactivation assay (ATTA) was performed as described in Spyropoulou *et al*., [47].

## Electronic supplementary material

Additional file 1: Figure S1: *Gene ontology (GO) and enzyme classifications (EC) for* S. lycopersicum *stem trichome transcriptome at level 2*. **(a)** Cellular component GO terms, **(b)** biological process GO terms, **(c)** molecular function GO terms and **(d)** general EC terms. **Figure S2.** T*ransactivation of terpene synthase promoters by 35S:RFP in* N. benthamiana *leaves*. Letters indicate significant differences (n = 4, ANOVA, *P* < 0.05 according to Tuckey’s B posthoc test). The normalized GUS activity of the SlTPS3, 7, 8 and 9 reporter constructs with the RFP effector construct is not significantly higher from the SlTPS5 reporter construct with the RFP effector construct, indicating that any relevant activation of an effector construct (in Figures 3, 4, and Additional file 1: Figure S3) must be significantly higher than that of the SlTPS5p:GUS reporter- 35S:RFP effector combination. **Figure S3.** T*ransactivation of terpene synthase promoters by SlWRKY78 and SlWRKY28 in* N. benthamiana *leaves*. Letters indicate significant differences (n = 3, ANOVA, *P* < 0.05 according to Tuckey’s B posthoc test). Representative results from two experiments are shown. The normalized GUS activity of the 35S:WRKY28 effector- SlTPS5p:GUS reporter construct combination was only marginally higher than that of the negative control (35S:RFP effector- SlTPS5p:GUS reporter constructs) and was not further investigated. **Figure S4.** *Nucleotide sequence of transcription factors* SlWRKY78 *(Solyc07g055280.2.1),* SlWRKY28 *(Solyc12g011200.1.1),* SlWRKY73 *(Solyc03g113120.2.1) and* SlMYC1 *(KF430611)*. The predicted coding sequences are in capital letters, 5′ and 3′ UTRs are in small letter type. Start and stop codons are in bold. **Table S1.** KEGG pathways found in the *S. lycopersicum* stem trichome transcriptome. **Table S2.** Selected regulatory motifs in the sequence of SlTPS5, 3 and 7 promoters analyzed by PLACE [65]. **Table S3.** List of primers used. (DOC 1 MB)

## Abbreviations

TPS: Terpene synthase
TF: Transcription factor
EOT1: Expression of terpenoids 1
SGN: Solanaceae genomics network
JA: Jasmonic acid.

## References

[1] Schilmiller AL, Last RL, Pichersky E (2008). Harnessing plant trichome biochemistry for the production of useful compounds. Plant J.

[2] Tissier A (2012). Glandular trichomes: what comes after expressed sequence tags?. Plant J.

[3] McDowell ET, Kapteyn J, Schmidt A, Li C, Kang JH, Descour A, Shi F, Larson M, Schilmiller A, An L, Jones AD, Pichersky E, Soderlund CA, Gang DR (2011). Comparative functional genomic analysis of Solanum glandular trichome types. Plant Physiol.

[4] Schilmiller A, Shi F, Kim J, Charbonneau AL, Holmes D, Daniel Jones A, Last RL (2010). Mass spectrometry screening reveals widespread diversity in trichome specialized metabolites of tomato chromosomal substitution lines. Plant J.

[5] Besser K, Harper A, Welsby N, Schauvinhold I, Slocombe S, Li Y, Dixon RA, Broun P (2009). Divergent regulation of terpenoid metabolism in the trichomes of wild and cultivated tomato species. Plant Physiol.

[6] Xie Z, Kapteyn J, Gang DR (2008). A systems biology investigation of the MEP/terpenoid and shikimate/phenylpropanoid pathways points to multiple levels of metabolic control in sweet basil glandular trichomes. Plant J.

[7] Gang DR, Wang J, Dudareva N, Nam KH, Simon JE, Lewinsohn E, Pichersky E (2001). An investigation of the storage and biosynthesis of phenylpropenes in sweet basil. Plant Physiol.

[8] Cui H, Zhang ST, Yang HJ, Ji H, Wang XJ (2011). Gene expression profile analysis of tobacco leaf trichomes. BMC Plant Biol.

[9] Harada E, Kim JA, Meyer AJ, Hell R, Clemens S, Choi YE (2010). Expression profiling of tobacco leaf trichomes identifies genes for biotic and abiotic stresses. Plant Cell Physiol.

[10] Lange BM, Wildung MR, Stauber EJ, Sanchez C, Pouchnik D, Croteau R (2000). Probing essential oil biosynthesis and secretion by functional evaluation of expressed sequence tags from mint glandular trichomes. Proc Natl Acad Sci U S A.

[11] Aziz N, Paiva NL, May GD, Dixon RA (2005). Transcriptome analysis of alfalfa glandular trichomes. Planta.

[12] Wang W, Wang Y, Zhang Q, Qi Y, Guo D (2009). Global characterization of Artemisia annua glandular trichome transcriptome using 454 pyrosequencing. BMC Genomics.

[13] Wang G, Tian L, Aziz N, Broun P, Dai X, He J, King A, Zhao PX, Dixon RA (2008). Terpene biosynthesis in glandular trichomes of hop. Plant Physiol.

[14] Luo H, Li Y, Sun C, Wu Q, Song J, Sun Y, Steinmetz A, Chen S (2010). Comparison of 454-ESTs from Huperzia serrata and Phlegmariurus carinatus reveals putative genes involved in lycopodium alkaloid biosynthesis and developmental regulation. BMC Plant Biol.

[15] Bleeker PM, Spyropoulou EA, Diergaarde PJ, Volpin H, De Both MT, Zerbe P, Bohlmann J, Falara V, Matsuba Y, Pichersky E, Haring MA, Schuurink RC (2011). RNA-seq discovery, functional characterization, and comparison of sesquiterpene synthases from Solanum lycopersicum and Solanum habrochaites trichomes. Plant Mol Biol.

[16] van Schie CC, Haring MA, Schuurink RC (2007). Tomato linalool synthase is induced in trichomes by jasmonic acid. Plant Mol Biol.

[17] Bleeker PM, Diergaarde PJ, Ament K, Guerra J, Weidner M, Schutz S, de Both MT, Haring MA, Schuurink RC (2009). The role of specific tomato volatiles in tomato-whitefly interaction. Plant Physiol.

[18] Schilmiller AL, Schauvinhold I, Larson M, Xu R, Charbonneau AL, Schmidt A, Wilkerson C, Last RL, Pichersky E (2009). Monoterpenes in the glandular trichomes of tomato are synthesized from a neryl diphosphate precursor rather than geranyl diphosphate. Proc Natl Acad Sci U S A.

[19] Kang JH, Liu G, Shi F, Jones AD, Beaudry RM, Howe GA (2010). The tomato odorless-2 mutant is defective in trichome-based production of diverse specialized metabolites and broad-spectrum resistance to insect herbivores. Plant Physiol.

[20] Bleeker PM, Mirabella R, Diergaarde PJ, VanDoorn A, Tissier A, Kant MR, Prins M, de Vos M, Haring MA, Schuurink RC (2012). Improved herbivore resistance in cultivated tomato with the sesquiterpene biosynthetic pathway from a wild relative. Proc Natl Acad Sci U S A.

[21] Falara V, Akhtar TA, Nguyen TT, Spyropoulou EA, Bleeker PM, Schauvinhold I, Matsuba Y, Bonini ME, Schilmiller AL, Last RL, Schuurink RC, Pichersky E (2011). The tomato terpene synthase gene family. Plant Physiol.

[22] Matsuba Y, Nguyen TT, Wiegert K, Falara V, Gonzales-Vigil E, Leong B, Schafer P, Kudrna D, Wing RA, Bolger AM, Usadel B, Tissier A, Fernie AR, Barry CS, Pichersky E (2013). Evolution of a complex locus for terpene biosynthesis in solanum. Plant Cell.

[23] De Geyter N, Gholami A, Goormachtig S, Goossens A (2012). Transcriptional machineries in jasmonate-elicited plant secondary metabolism. Trends Plant Sci.

[24] Yang CQ, Fang X, Wu XM, Mao YB, Wang LJ, Chen XY (2012). Transcriptional regulation of plant secondary metabolism. J Integr Plant Biol.

[25] van der Fits L, Memelink J (2000). ORCA3, a jasmonate-responsive transcriptional regulator of plant primary and secondary metabolism. Science.

[26] Zhang H, Hedhili S, Montiel G, Zhang Y, Chatel G, Pre M, Gantet P, Memelink J (2011). The basic helix-loop-helix transcription factor CrMYC2 controls the jasmonate-responsive expression of the ORCA genes that regulate alkaloid biosynthesis in Catharanthus roseus. Plant J.

[27] Suttipanta N, Pattanaik S, Kulshrestha M, Patra B, Singh SK, Yuan L (2011). The transcription factor CrWRKY1 positively regulates the terpenoid indole alkaloid biosynthesis in catharanthus roseus. Plant Physiol.

[28] Xu YH, Wang JW, Wang S, Wang JY, Chen XY (2004). Characterization of GaWRKY1, a cotton transcription factor that regulates the sesquiterpene synthase gene (+)-delta-cadinene synthase-A. Plant Physiol.

[29] Ma D, Pu G, Lei C, Ma L, Wang H, Guo Y, Chen J, Du Z, Wang H, Li G, Ye H, Liu B (2009). Isolation and characterization of AaWRKY1, an Artemisia annua transcription factor that regulates the amorpha-4,11-diene synthase gene, a key gene of artemisinin biosynthesis. Plant Cell Physiol.

[30] Yu ZX, Li JX, Yang CQ, Hu WL, Wang LJ, Chen XY (2012). The jasmonate-responsive AP2/ERF transcription factors AaERF1 and AaERF2 positively regulate artemisinin biosynthesis in Artemisia annua L. Mol Plant.

[31] Lu X, Zhang L, Zhang F, Jiang W, Shen Q, Zhang L, Lv Z, Wang G, Tang K (2013). AaORA, a trichome-specific AP2/ERF transcription factor of Artemisia annua, is a positive regulator in the artemisinin biosynthetic pathway and in disease resistance to Botrytis cinerea. New Phytol.

[32] Lorenzo O, Chico JM, Sanchez-Serrano JJ, Solano R (2004). JASMONATE-INSENSITIVE1 encodes a MYC transcription factor essential to discriminate between different jasmonate-regulated defense responses in Arabidopsis. Plant Cell.

[33] Hong GJ, Xue XY, Mao YB, Wang LJ, Chen XY (2012). Arabidopsis MYC2 interacts with DELLA proteins in regulating sesquiterpene synthase gene expression. Plant Cell.

[34] Kant MR, Ament K, Sabelis MW, Haring MA, Schuurink RC (2004). Differential timing of spider mite-induced direct and indirect defenses in tomato plants. Plant Physiol.

[35] Ament K, Kant MR, Sabelis MW, Haring MA, Schuurink RC (2004). Jasmonic acid is a key regulator of spider mite-induced volatile terpenoid and methyl salicylate emission in tomato. Plant Physiol.

[36] **Blast2Go**. http://www.blast2go.com

[37] Conesa A, Gotz S, Garcia-Gomez JM, Terol J, Talon M, Robles M (2005). Blast2GO: a universal tool for annotation, visualization and analysis in functional genomics research. Bioinformatics.

[38] Li L, Zhao Y, McCaig BC, Wingerd BA, Wang J, Whalon ME, Pichersky E, Howe GA (2004). The tomato homolog of CORONATINE-INSENSITIVE1 is required for the maternal control of seed maturation, jasmonate-signaled defense responses, and glandular trichome development. Plant Cell.

[39] Thines B, Katsir L, Melotto M, Niu Y, Mandaokar A, Liu G, Nomura K, He SY, Howe GA, Browse J (2007). JAZ repressor proteins are targets of the SCF(COI1) complex during jasmonate signalling. Nature.

[40] Katsir L, Schilmiller AL, Staswick PE, He SY, Howe GA (2008). COI1 is a critical component of a receptor for jasmonate and the bacterial virulence factor coronatine. Proc Natl Acad Sci U S A.

[41] Tholl D, Lee S (2011). Terpene specialized metabolism in Arabidopsis thaliana. Arabidopsis Book.

[42] Sapir-Mir M, Mett A, Belausov E, Tal-Meshulam S, Frydman A, Gidoni D, Eyal Y (2008). Peroxisomal localization of Arabidopsis isopentenyl diphosphate isomerases suggests that part of the plant isoprenoid mevalonic acid pathway is compartmentalized to peroxisomes. Plant Physiol.

[43] Simkin AJ, Guirimand G, Papon N, Courdavault V, Thabet I, Ginis O, Bouzid S, Giglioli-Guivarc’h N, Clastre M (2011). Peroxisomal localisation of the final steps of the mevalonic acid pathway in planta. Planta.

[44] Thabet I, Guirimand G, Courdavault V, Papon N, Godet S, Dutilleul C, Bouzid S, Giglioli-Guivarc’h N, Clastre M, Simkin AJ (2011). The subcellular localization of periwinkle farnesyl diphosphate synthase provides insight into the role of peroxisome in isoprenoid biosynthesis. J Plant Physiol.

[45] Spyropoulou EA (2012). PhD thesis. Transcription Factors Regulating Terpene Synthases in Tomato Trichomes.

[46] **The GENSCAN Web Server at MIT**. http://genes.mit.edu/GENSCAN.html

[47] Spyropoulou EA, Haring MA, Schuurink RC (2014). Expression of Terpenoids 1, a glandular trichome-specific transcription factor from tomato that activates the Terpene Synthase 5 promoter. Plant Mol Biol.

[48] Walter MH, Hans J, Strack D (2002). Two distantly related genes encoding 1-deoxy-d-xylulose 5-phosphate synthases: differential regulation in shoots and apocarotenoid-accumulating mycorrhizal roots. Plant J.

[49] Paetzold H, Garms S, Bartram S, Wieczorek J, Uros-Gracia EM, Rodriguez-Concepcion M, Boland W, Strack D, Hause B, Walter MH (2010). The isogene 1-deoxy-D-xylulose 5-phosphate synthase 2 controls isoprenoid profiles, precursor pathway allocation, and density of tomato trichomes. Mol Plant.

[50] Sanchez-Hernandez C, Lopez MG, Delano-Frier JP (2006). Reduced levels of volatile emissions in jasmonate-deficient spr2 tomato mutants favour oviposition by insect herbivores. Plant Cell Environ.

[51] Okada A, Shimizu T, Okada K, Kuzuyama T, Koga J, Shibuya N, Nojiri H, Yamane H (2007). Elicitor induced activation of the methylerythritol phosphate pathway toward phytoalexins biosynthesis in rice. Plant Mol Biol.

[52] Oudin A, Mahroug S, Courdavault V, Hervouet N, Zelwer C, Rodriguez-Concepcion M, St-Pierre B, Burlat V (2007). Spatial distribution and hormonal regulation of gene products from methyl erythritol phosphate and monoterpene-secoiridoid pathways in Catharanthus roseus. Plant Mol Biol.

[53] Arimura G, Garms S, Maffei M, Bossi S, Schulze B, Leitner M, Mithofer A, Boland W (2008). Herbivore-induced terpenoid emission in Medicago truncatula: concerted action of jasmonate, ethylene and calcium signaling. Planta.

[54] Kim YB, Kim SM, Kang MK, Kuzuyama T, Lee JK, Park SC, Shin SC, Kim SU (2009). Regulation of resin acid synthesis in Pinus densiflora by differential transcription of genes encoding multiple 1-deoxy-D-xylulose 5-phosphate synthase and 1-hydroxy-2-methyl-2-(E)-butenyl 4-diphosphate reductase genes. Tree Physiol.

[55] Yang Z, Park H, Lacy GH, Cramer CL (1991). Differential activation of potato 3-hydroxy-3-methylglutaryl coenzyme A reductase genes by wounding and pathogen challenge. Plant Cell.

[56] Choi D, Bostock RM, Avdiushko S, Hildebrand DF (1994). Lipid-derived signals that discriminate wound- and pathogen-responsive isoprenoid pathways in plants: methyl jasmonate and the fungal elicitor arachidonic acid induce different 3-hydroxy-3-methylglutaryl-coenzyme A reductase genes and antimicrobial isoprenoids in Solanum tuberosum L. Proc Natl Acad Sci U S A.

[57] Ha SH, Kim JB, Hwang YS, Lee SW (2003). Molecular characterization of three 3-hydroxy-3-methylglutaryl-CoA reductase genes including pathogen-induced Hmg2 from pepper (Capsicum annuum). Biochim Biophys Acta.

[58] Hui D, Iqbal J, Lehmann K, Gase K, Saluz HP, Baldwin IT (2003). Molecular interactions between the specialist herbivore Manduca sexta (lepidoptera, sphingidae) and its natural host Nicotiana attenuata: V. microarray analysis and further characterization of large-scale changes in herbivore-induced mRNAs. Plant Physiol.

[59] Kondo K, Uritani I, Oba K (2003). Induction mechanism of 3-hydroxy-3-methylglutaryl-CoA reductase in potato tuber and sweet potato root tissues. Biosci Biotechnol Biochem.

[60] Bede JC, Musser RO, Felton GW, Korth KL (2006). Caterpillar herbivory and salivary enzymes decrease transcript levels of Medicago truncatula genes encoding early enzymes in terpenoid biosynthesis. Plant Mol Biol.

[61] Ament K, Van Schie CC, Bouwmeester HJ, Haring MA, Schuurink RC (2006). Induction of a leaf specific geranylgeranyl pyrophosphate synthase and emission of (E, E)-4,8,12-trimethyltrideca-1,3,7,11-tetraene in tomato are dependent on both jasmonic acid and salicylic acid signaling pathways. Planta.

[62] Akhtar TA, Matsuba Y, Schauvinhold I, Yu G, Lees HA, Klein SE, Pichersky E (2012). The tomato cis-prenyltransferase gene family. Plant J.

[63] **TAIR10 genome release**. http://arabidopsis.org

[64] Fernandez-Calvo P, Chini A, Fernandez-Barbero G, Chico JM, Gimenez-Ibanez S, Geerinck J, Eeckhout D, Schweizer F, Godoy M, Franco-Zorrilla JM, Pauwels L, Witters E, Puga MI, Paz-Ares J, Goossens A, Reymond P, De Jaeger G, Solano R (2011). The Arabidopsis bHLH transcription factors MYC3 and MYC4 are targets of JAZ repressors and act additively with MYC2 in the activation of jasmonate responses. Plant Cell.

[65] **PLACE**. http://www.dna.affrc.go.jp/PLACE/

[66] **MEME**. http://meme.sdsc.edu/meme/intro.html

[67] Singh KB (1998). Transcriptional regulation in plants: the importance of combinatorial control. Plant Physiol.

[68] Abe H, Yamaguchi-Shinozaki K, Urao T, Iwasaki T, Hosokawa D, Shinozaki K (1997). Role of Arabidopsis MYC and MYB homologs in drought- and abscisic acid-regulated gene expression. Plant Cell.

[69] Abe H, Urao T, Ito T, Seki M, Shinozaki K, Yamaguchi-Shinozaki K (2003). Arabidopsis AtMYC2 (bHLH) and AtMYB2 (MYB) function as transcriptional activators in abscisic acid signaling. Plant Cell.

[70] **CLCbio bioinformatics software**. http://www.clcbio.com/

[71] Quevillon E, Silventoinen V, Pillai S, Harte N, Mulder N, Apweiler R, Lopez R (2005). InterProScan: protein domains identifier. Nucleic Acids Res.

[72] Ogata H, Goto S, Fujibuchi W, Kanehisa M (1998). Computation with the KEGG pathway database. Biosystems.

[73] **PASW Statistics 17.0**. http://www.spss.com

[74] Huang S, Gao Y, Liu J, Peng X, Niu X, Fei Z, Cao S, Liu Y (2012). Genome-wide analysis of WRKY transcription factors in Solanum lycopersicum. Mol Genet Genomics.

[75] van Engelen FA, Molthoff JW, Conner AJ, Nap JP, Pereira A, Stiekema WJ (1995). pBINPLUS: an improved plant transformation vector based on pBIN19. Transgenic Res.

