# Supplementary material for: RNA sequencing on Solanum lycopersicum trichomes identifies transcription factors that activate terpene synthase promoters
Source: BMC Genomics. 2014 May 27;15(1):402. doi: 10.1186/1471-2164-15-402 (PMC4041997; doi:10.1186/1471-2164-15-402)
Supplement: Supplementary file 1 — Additional file 1: Figure S1: Gene ontology (GO) and enzyme classifications (EC) for S. lycopersicum stem trichome transcriptome at level 2. (a) Cellular component GO terms, (b) biological process GO terms, (c) molecular function GO terms and (d) general EC terms. Figure S2. Transactivation of terpene synthase promoters by 35S:RFP in N. benthamiana leaves. Letters indicate significant differences (n = 4, ANOVA, P < 0.05 according to Tuckey’s B posthoc test). The normalized GUS activity of the SlTPS3, 7, 8 and 9 reporter constructs with the RFP effector construct is not significantly higher from the SlTPS5 reporter construct with the RFP effector construct, indicating that any relevant activation of an effector construct (in Figures 3, 4, and Additional file 1: Figure S3) must be significantly higher than that of the SlTPS5p:GUS reporter- 35S:RFP effector combination. Figure S3. Transactivation of terpene synthase promoters by SlWRKY78 and SlWRKY28 in N. benthamiana leaves. Letters indicate significant differences (n = 3, ANOVA, P < 0.05 according to Tuckey’s B posthoc test). Representative results from two experiments are shown. The normalized GUS activity of the 35S:WRKY28 effector- SlTPS5p:GUS reporter construct combination was only marginally higher than that of the negative control (35S:RFP effector- SlTPS5p:GUS reporter constructs) and was not further investigated. Figure S4. Nucleotide sequence of transcription factors SlWRKY78 (Solyc07g055280.2.1), SlWRKY28 (Solyc12g011200.1.1), SlWRKY73 (Solyc03g113120.2.1) and SlMYC1 (KF430611). The predicted coding sequences are in capital letters, 5′ and 3′ UTRs are in small letter type. Start and stop codons are in bold. Table S1. KEGG pathways found in the S. lycopersicum stem trichome transcriptome. Table S2. Selected regulatory motifs in the sequence of SlTPS5, 3 and 7 promoters analyzed by PLACE [65]. Table S3. List of primers used. (DOC 1 MB) [file 12864_2014_6088_MOESM1_ESM.doc]

**Figure S1**


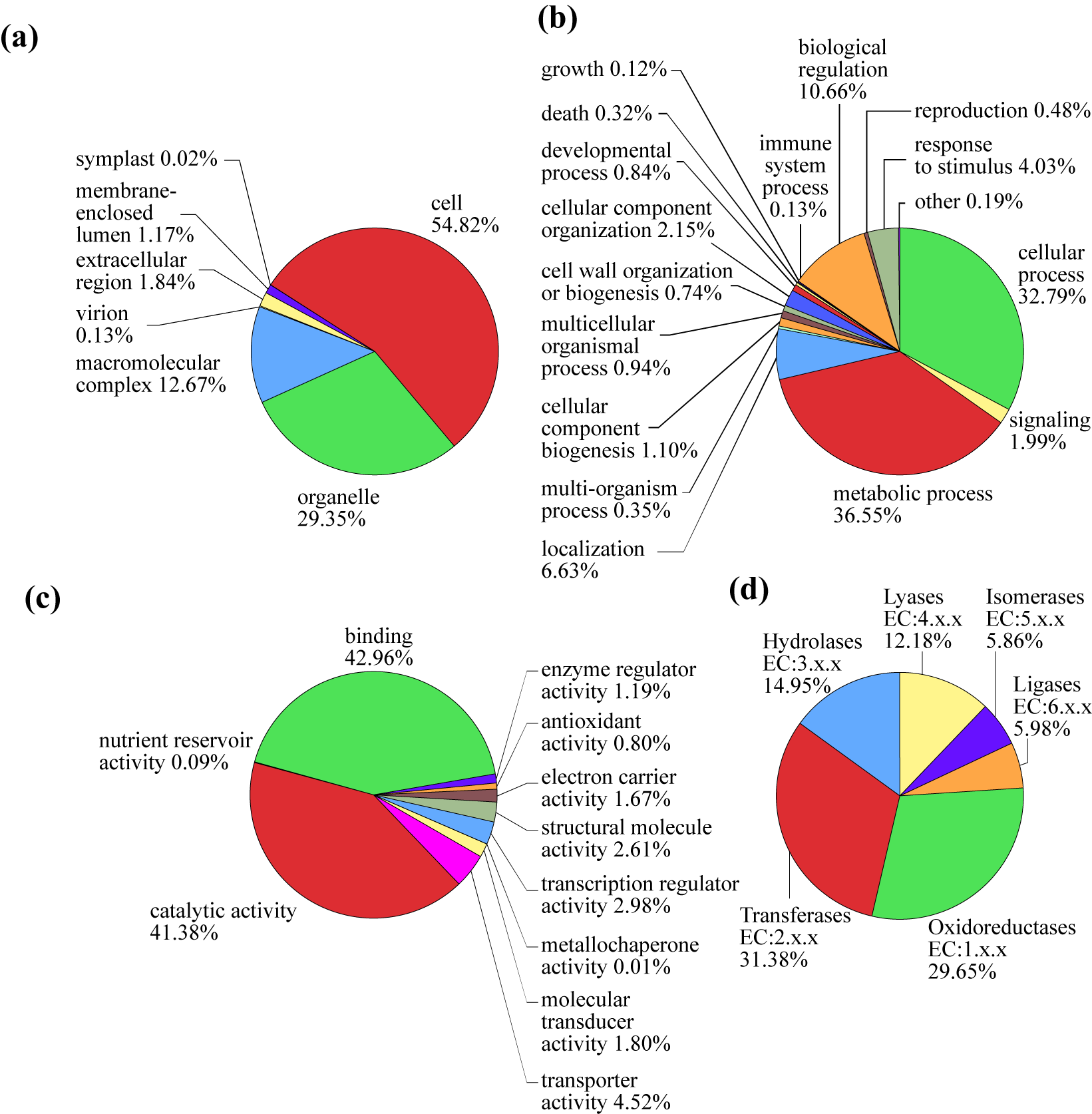


**Figure S2**


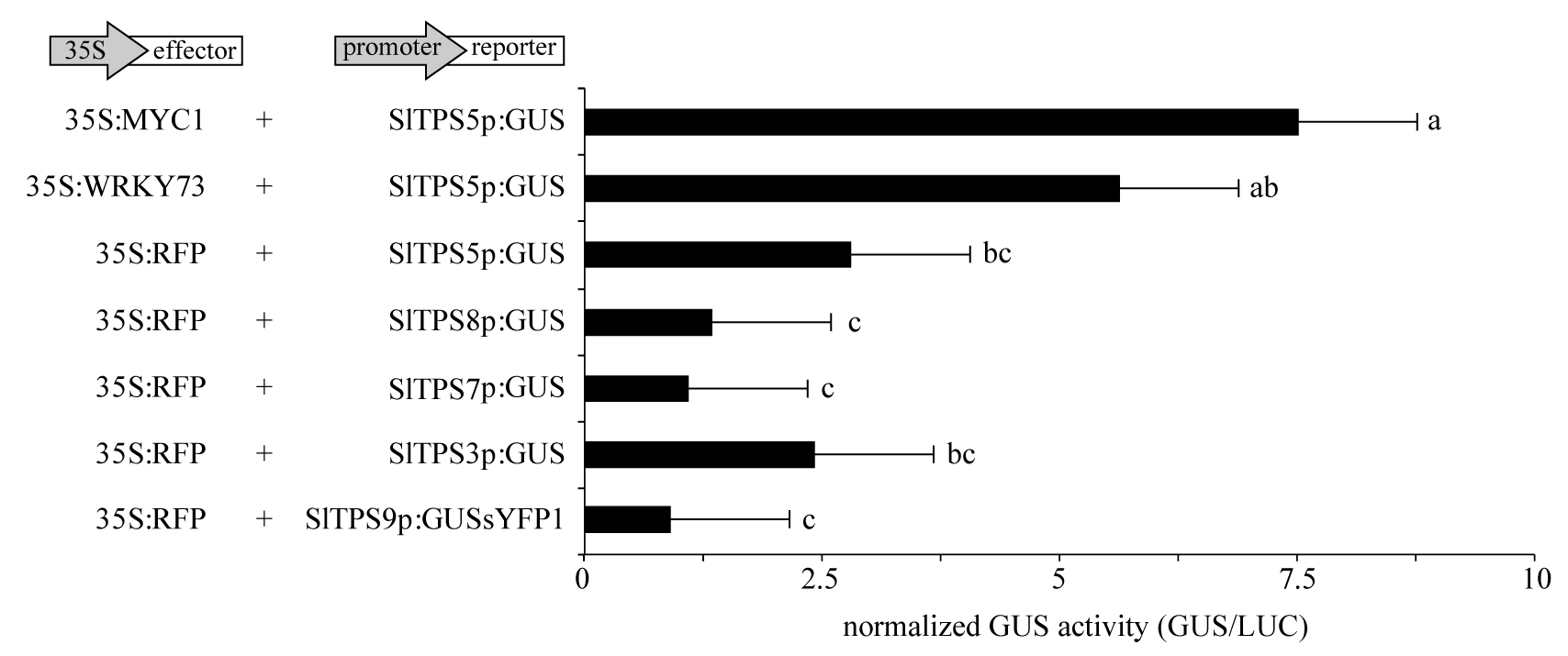


**Figure S3**

**
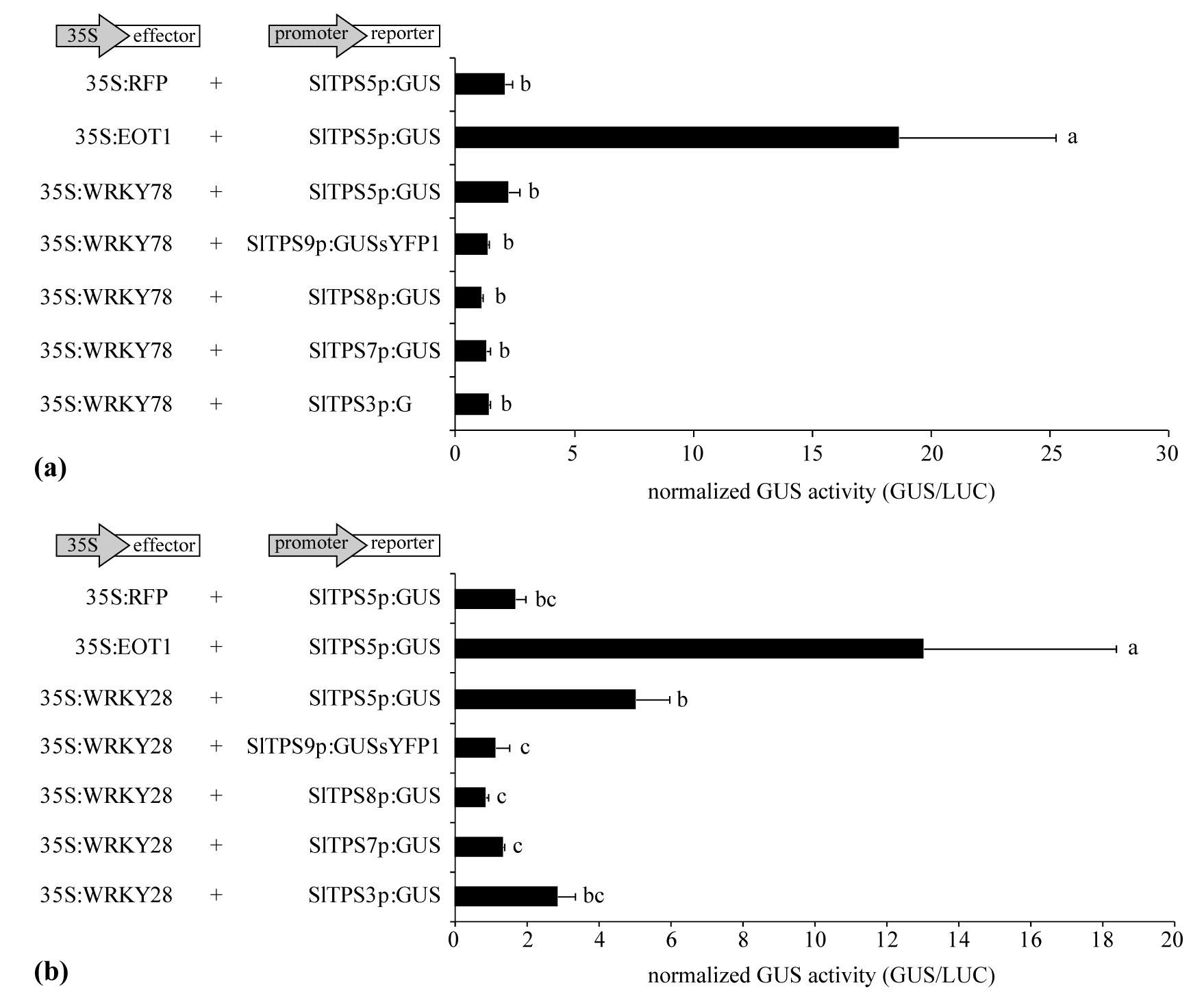
**

**Figure S4**

*SlMYC1*

ctcacctaacaaacaaaatctctcattttctgttttttgtaaaattcttcaatttaattga**ATG**ACGGACTATAGATTATGGAGTAATACCAATACTACTAATACATGTGATGATACTATGATGATGGATTCTTTTTTATCTTCCGATCCATCCTCTTTTTGGCCTGCTTCCACTCCCAATCGTCCGACTCCGGTGAACGGAGTCGGAGAAACGATGCCGTTTTTCAATCAAGAATCACTACAGCAAAGGCTTCAGGCTTTAATTGACGGTGCTCGTGAATCATGGGCATATGCTATTTTCTGGCAATCGTCAGTTGTTGATTTTGCGAGCCAAACTGTATTGGGTTGGGGAGATGGGTATTATAAAGGAGAAGAAGATAAGAATAAACGGAGAGGGTCGTCTAGTTCAGCAGCTAATTTTGTTGCTGAGCAAGAGCATAGAAAGAAGGTGCTTCGGGAGCTGAATTCATTAATATCCGGTGTACAAGCTTCCGCCGGAAACGGAACTGATGATGCAGTGGATGAGGAAGTGACGGATACTGAATGGTTTTTTCTGATTTCAATGACCCAATCGTTTGTTAACGGTAACGGGCTTCCGGGCTTGGCGATGTACAGTTCAAGCCCAATTTGGGTTACTGGAACAGAGAAATTAGCTGCTTCTCAATGTGAACGGGCCAGGCAAGCCCAAGGTTTCGGGCTTCAGACGATTGTGTGTATTCCTTCAGCTAACGGTGTAGTGGAGCTTGGTTCGACTGAGCTGATATTCCAAAGCTCGGATTTGATGAACAAGGTTAAGTATTTGTTTAACTTCAATATTGATATGGGGTCTGTTACAGGCTCAGGTTCGGGCTCAGGCTCTTGTGCTGTGCATCCTGAGCCCGATCCTTCGGCCCTTTGGCTTACGGATCCATCTTCCTCGGTTGTGGAACCTAAGGATTCGTTAATTCATAGTAGTAGTAGGGATGTTCAACTTGTGTATGGAAATGAGAATTCTGAAAATCAGCAGCAGCATTGTCAAGGATTTTTCACAAAGGAGTTGAATTTTTCGGGTTATGGATTTGATGGAAGTAGTAATAGGAATAAAACTGGAATTTCTTGTAAGCCGGAGTCCAGGGAGATATTGAATTTTGGTGATAGTAGTAAGAGATTTTCAGGGCAATCACAGTTGGGTCCTGGGCCTGGGCTCATGGAGGAGAACAAGAACAAGAACAAGAACAAGAAAAGGTCACTTGGATCAAGGGGAAACAATGAAGAAGGAATGCTTTCGTTTGTTTCGGGTGTGATCTTGCCAACTTCAACA

ATGGGGAAGTCCGGGGATTCTGATCACTCAGATCTCGAAGCCTCAGTGGTGAAGGAGGCCGTTGTAGAACCTGAAAAGAAGCCGAGGAAGCGAGGGAGGAAACCAGCCAATGGAAGGGAGGAGCCATTGAATCACGTGGAAGCGGAGAGACAGAGGAGGGAGAAATTGAATCAAAGATTCTACGCGCTCAGAGCCGTAGTCCCAAATGTGTCTAAAATGGATAAGGCATCACTTCTTGGAGATGCAATTGCATACATCAATGAGTTGAAATCAAAAGTTCAAAATTCAGATTTAGATAAAGAGGAGTTGAGGAGCCAAATTGAATGTTTAAGGAAGGAATTAACCAACAAGGGATCATCAAACTATTCCGCCTCCCCTCCATTGAATCAAGATGTCAAGATTGTCGATATGGACATTGACGTTAAGGTGATTGGATGGGATGCTATGATTCGTATACAATGTAGTAAAAAGAACCATCCAGCTGCCAGGCTAATGGCAGCCCTCAAGGACTTGGACCTAGACGTGCACCACGCTAGTGTTTCCGTGGTGAATGATTTGATGATCCAACAAGCCACAGTCAAAATGGGGAGCCGGCTTTATGCTCAAGAACAGCTTAGGATAGCATTGACATCAAAAATTGCTGAATCGCGA**TGA**aattatgtccctagtgagctatgtataatgttatcttctaatgagcgagaattttcttctctgtatataaatgtgatgaaaccaatactagagatctcgagttgaggctttttagttcatgtaagattagatatatatatatgatgcagcttcatccttttgtattcttcatccaggaaataaatgagaaaccaataattggtggctgatggtcaacttc

*SlWRKY78*

actacctttccccacttcataccaactctttcttagcaaactttttattttctctcattttttcttctaatttctatatgatataaaacaaaatttagttaatcctcaaaaagttttattttctttcttttttttttaagttatataagttatttttgttgttttttggttc**ATG**GAAGATAGTTCATACAAAAATACTATTTTTTTATAAACAAGAAGATTCCACCGGAACTCCACCGGATAATGCTGCTGATTCTTGTTTTTCCGGTGATGAAGCGGCGGAAGTTAGCATGCCATCACCTAGAAAAAGTAGGAGAGGAGCCAAAAAGAAAGTAATTTCAGTGCCAATAATTGAAGGTGATGGATCAAGAAGTAAAGGGGAAGTTTATCCACCACAAGATTCTTGGTCTTGGAGAAAATATGGACAAAAACCAATTAAAGGATCACCTTATCCCAGGGGATATTATCGATGCAGTAGCTCCAAAGGCTGTCCCGCCAGAAAACAAGTCGAGCGTAGCCGCCTGGACCCCACCATGCTTCTCATTACCTATTGCTCCGAACACAATCACCAAATCCCGGCCGCCGCCGCCGCCAAACACCACCATCACAACCACCCTACTACCACCACCAGTTCACCCACTACCTCTACCGGCACCGCCGGAGACAATAATGCTACTGCGGCTGTAGCCACGGACTCTACCGTAGTAGACAAATCATCCCCCGAAGAACCAGATCTATTTGCTTATCAACACGATAATGGATTTTCAGAGCTCGCCGGTGAGTTAGGTTGGTTTTCCGATATGGGAACAACAACTACGTTTATGGAGAGTACGTCGTCGTCCATGGCGGGATCCACGTGGAACGACAGTGACGTGGCGTTAATGTTGCCGATTCGAGAAGAGGATCAGTCGTTGTACGGTGATCTCGGTGAATTACCGGAATGTTCAGTTGTTTTCCGGCGGTACAGTGTGGAAACTCCCTGCTGCGGAGGTACAGGA**TAA**caaatcatttgtcaatttttcactgaaattgccatatttttattttttaattatcttcctgacttttggttccacgttcttcgtgaccctttttttaa

*SlWRKY28*

acttcataacgagtgccctcaaattcatattttcttcgccaaggaccatccatttgctattacctatcaagctacatactcttttttttttctctctccaaaacatttctcatctatcatatctatttttttattgcacaaaactatagtgttaatgtaatttaagaattcgaaataatatttttctccaat**ATG**TCTGATAATCCTTTTTATCATGATTACATGGGAATTGGTGGAGGGATCAATAATACATTCCCTTTTTTGGTTGAAAATCCTTCAAATTATAATAACCAACCAATAACTCCAAATATTCAAAATCAACAATTTGTTCCTTCTTCTTATATGACTCTCACTGAGTGTTTACATGGCTCTATGGACTACAACACACTATCAAATGCTTTTGGTATGTCTTGTTCATCATCATCTGAAGTTGTTTGTCCACATATCGATAATCAAGACTCTACTGAAAAATATAACGTCTCTACTACTGCAGAAGAACCTGTCTTAGATTCACCTCTAGGAGATAATCAAACGAGCGTTGAAGTTCCACCAACGCCAAATTCTTCGATATCTTCTACTTCTAATGAGGTTGGAGAGCAAGAAGATTCTTCCAAAATCAAGAAACATGTTCAAGTAAAAGGGAGCCAAGAAGAAGGAGAAGACAAGTCTAAGAAAGAGTGCATAGCAAAAAAGAAAGGAGAAAAAAAGATAAAAGAACCAAGATTTGCCTTCATGACAAAGAGTGAGATTGATAATCTTGAAGATGGTTATCGATGGAGAAAATATGGACAAAAGGCAGTGAAGAACAGCCCTTTTCCTAGGAACTATTATAAATGCACAACTCAAAAGTGCAATGTGAAGAAACGTGTTGAAAGGTCATATGAAGATTCATCAATTGTGATAACAACATATGAAGGCCAACACAATCATCATTGTCCAGCAACTCTTAGGGGAAATGCATCTTTTTTATCTTCATCACATTTTATGCCTAATTTTCCTCCACAACTATTTTCCCAAATGCTAAATATTCCACCAAACAACCAAAATCTCCTCATTACTTCTTCGACCTATAATTATAATAATAATAATAACAATTATTATTATCAACAACAACAACATCAAGGTTCAGAATATACCCTATTTGGTGGAGGCACAAATAATGGTGATGCTTCATGGGTCCAAAAACAAGAGCCATCT**TAG**ttcactacaaggttttcgtgacgaatcgactattcttatccatcaagaatttttgtacaagtttcttgtagcattgactatttttatatatacagacatggttgaatttaatttgaatttcatggt

*SlWRKY73*

**ATG**GAAGAAGTAATGGAAGAAAATCAAAGGCTAAAGAAACACTTAGATAAAATCATGAAGGATTATCGGAATCTTCAAATGCAATTCCATGAAGTTGCACAAAGAGATGCTGAAAAAACTAATACTGATGTTAAACATGACGAAGCTGAACTTGTTTCCCTTAGCCTAGGAAGGACTTCAAGTGACACAAAAAAGGAGTTATCCAAATTAATTTTGAGCAAAAAAGAGAATGATGAGAAGGAAGAAGATAACCTAACCCTAGCATTAGATTGCAAGTTTCAATCATCGACCAAATCTTCACCATCAAATCTCAGTCCAGAGAATAGTTTAGGAGAAGTGAAGGACGACGAAAAAGGAACTGATCAAACATGGCCACCTCACAAAGTTCTCAAGACGATAAGGAATGAAGAAGATGATGTGACACAACAAAACCCTACTAAAAGAGCAAAGGTTTCCGTCAGAGTTAGATGTGACACCCCAACGATGAACGATGGATGTCAATGGAGAAAATATGGACAAAAAATTGCAAAAGGGAACCCATGTCCTAGAGCTTACTACCGTTGCACGGTAGCACCAAATTGCCCCGTAAGAAAACAGGTACAAAGATGCATTCAAGACATGTCTATATTGATCACAACATACGAAGGAACACATAACCATCCACTTCCTCATTCAGCCACATCAATGGCTTTCACCACTTCAGCAGCCGCTTCCATGCTATTATCCGGTTCATCCAGCTCCGGATCGGGCCCCACAAGTAGTACAGCCTCTGCCACAACCTCTGCGCTCAACTATTGCTTCTCTGATAACTCAAAACCAAACCCATTTTATAACCTTCCACATTCATCCATTTCTTCATCATCACATTCTCAGTACCCTACAATCACTCTTGATTTAACATCAAACTCGTCCACTTCCTCATTTCCTGGCCAAAATTATAGAACAATCGCGAATAGTAATAATTATCCCCCGCGATATAATAATAATAATTCATCCACAAATATTCTCAATTTCAGTTCCTTTGAATCTAATCATCTTCTTCCTATGTCTTGGAGTAACAGAAATAACCAAGACACCCATTCCCAGTCTTATCTACAAAACAATATAAAAAGCGCGGCATCTACACAGACTTTATTACCACAAGACACAATTGCAGCTGCAACAAAAGCAATAACATCAGATCCGAAGTTTCAATCTGCATTGGCTGTTGCTCTTACATCTATCATTGGCTCACGCAGCGGAAATCATCATATTGACGAAAAATCTGGGCAGAATATGAAGGTTACTGAGCCATTTCCAGTGCTTTGTAGCTTCCCATCCACCTCTCCTGGTGATCACAAAGATTACACACTG**TGA**ttcattttaatttctttagttagagtttttgcttgtacgttacaagtaaaaaactggtatcagcgttattaatcaggttgtatttttttaatgatttggagaatatgtagtaccaaaatgtagatattttagtatttagtgaattaattgacgacttcgt

**Table S1.** KEGG pathways found in the *S. lycopersicum* stem trichome transcriptome. The classification of the EC numbers in KEGG pathways was performed via Blast2GO (www.blast2go.com)

| **KEGG pathway** | **EC number** | **Enzyme Name** | **Nr of contigs** |
| --- | --- | --- | --- |
| **Photosynthesis** | ec:1.18.1.2 | ferredoxin---NADP+ reductase | 3 |
| **Carbon fixation** | ec:2.7.1.19 | phosphoribulokinase | 1 |
|  | ec:4.1.1.49 | phosphoenolpyruvate carboxykinase (ATP) | 1 |
|  | ec:4.1.1.39 | ribulose-bisphosphate carboxylase | 7 |
|  | ec:4.1.1.31 | phosphoenolpyruvate carboxylase | 7 |
|  | ec:5.3.1.6 | ribose-5-phosphate isomerase | 2 |
|  | ec:5.3.1.1 | triose-phosphate isomerase | 3 |
|  | ec:5.1.3.1 | ribulose-phosphate 3-epimerase | 3 |
|  | ec:2.2.1.1 | transketolase | 2 |
|  | ec:3.1.3.11 | fructose-bisphosphatase | 5 |
|  | ec:2.7.2.3 | phosphoglycerate kinase | 2 |
|  | ec:1.1.1.82 | malate dehydrogenase (NADP+) | 2 |
|  | ec:4.1.2.13 | fructose-bisphosphate aldolase | 4 |
|  | ec:1.1.1.39 | malate dehydrogenase (decarboxylating) | 2 |
|  | ec:1.1.1.37 | malate dehydrogenase | 7 |
|  | ec:2.6.1.1 | aspartate transaminase | 1 |
|  | ec:1.2.1.13 | glyceraldehyde-3-phosphate dehydrogenase (NADP+) (phosphorylating) | 2 |
|  | ec:2.7.1.40 | pyruvate kinase | 4 |
|  | ec:6.3.4.3 | formate---tetrahydrofolate ligase | 1 |
|  | ec:4.1.1.31 | phosphoenolpyruvate carboxylase | 7 |
|  | ec:1.5.1.20 | methylenetetrahydrofolate reductase [NAD(P)H] | 1 |
|  | ec:6.4.1.2 | acetyl-CoA carboxylase | 7 |
|  | ec:3.5.4.9 | methenyltetrahydrofolate cyclohydrolase | 2 |
|  | ec:2.3.3.8 | ATP citrate synthase | 6 |
|  | ec:6.2.1.5 | succinate---CoA ligase (ADP-forming) | 7 |
|  | ec:6.2.1.1 | acetate---CoA ligase | 2 |
|  | ec:1.1.1.42 | isocitrate dehydrogenase (NADP+) | 4 |
|  | ec:1.1.1.37 | malate dehydrogenase | 7 |
|  | ec:1.1.1.35 | 3-hydroxyacyl-CoA dehydrogenase | 1 |
|  | ec:1.5.1.5 | methylenetetrahydrofolate dehydrogenase (NADP+) | 2 |
|  | ec:4.2.1.3 | aconitate hydratase | 2 |
|  | ec:4.2.1.2 | fumarate hydratase | 3 |
| **Fructose and mannose metabolism** | ec:2.7.1.11 | 6-phosphofructokinase | 10 |
|  | ec:5.3.1.8 | mannose-6-phosphate isomerase | 3 |
|  | ec:5.3.1.5 | xylose isomerase | 1 |
|  | ec:5.3.1.1 | triose-phosphate isomerase | 3 |
|  | ec:2.7.1.4 | fructokinase | 3 |
|  | ec:2.7.1.1 | hexokinase | 1 |
|  | ec:3.2.1.78 | mannan endo-1,4-beta-mannosidase | 1 |
|  | ec:3.1.3.46 | fructose-2,6-bisphosphate 2-phosphatase | 1 |
|  | ec:3.1.3.11 | fructose-bisphosphatase | 5 |
|  | ec:2.7.7.22 | mannose-1-phosphate guanylyltransferase (GDP) | 1 |
|  | ec:4.1.2.13 | fructose-bisphosphate aldolase | 4 |
|  | ec:3.6.1.21 | ADP-sugar diphosphatase | 1 |
|  | ec:2.7.1.105 | 6-phosphofructo-2-kinase | 1 |
|  | ec:1.1.1.21 | aldehyde reductase | 1 |
|  | ec:2.7.1.90 | diphosphate---fructose-6-phosphate 1-phosphotransferase | 6 |
|  | ec:5.4.2.8 | phosphomannomutase | 1 |
|  | ec:2.7.1.52 | fucokinase | 1 |
| **Galactose metabolism** | ec:2.7.1.11 | 6-phosphofructokinase | 10 |
|  | ec:2.7.1.6 | galactokinase | 2 |
|  | ec:2.7.1.2 | glucokinase | 1 |
|  | ec:2.7.1.1 | hexokinase | 1 |
|  | ec:5.1.3.2 | UDP-glucose 4-epimerase | 4 |
|  | ec:3.2.1.26 | beta-fructofuranosidase | 3 |
|  | ec:3.2.1.23 | beta-galactosidase | 20 |
|  | ec:3.2.1.22 | alpha-galactosidase | 4 |
|  | ec:3.2.1.20 | alpha-glucosidase | 6 |
|  | ec:2.7.7.9 | UTP---glucose-1-phosphate uridylyltransferase | 1 |
|  | ec:2.7.7.12 | UDP-glucose---hexose-1-phosphate uridylyltransferase | 1 |
|  | ec:2.4.1.67 | galactinol---raffinose galactosyltransferase | 2 |
|  | ec:1.1.1.21 | aldehyde reductase | 1 |
|  | ec:5.4.2.2 | phosphoglucomutase | 3 |
| **Pentose phosphate pathway** | ec:2.7.1.15 | ribokinase | 7 |
|  | ec:2.7.1.12 | gluconokinase | 1 |
|  | ec:2.7.1.11 | 6-phosphofructokinase | 10 |
|  | ec:2.7.6.1 | ribose-phosphate diphosphokinase | 1 |
|  | ec:5.3.1.9 | glucose-6-phosphate isomerase | 3 |
|  | ec:5.3.1.6 | ribose-5-phosphate isomerase | 2 |
|  | ec:5.1.3.1 | ribulose-phosphate 3-epimerase | 3 |
|  | ec:3.1.1.31 | 6-phosphogluconolactonase | 3 |
|  | ec:2.2.1.2 | transaldolase | 1 |
|  | ec:2.2.1.1 | transketolase | 2 |
|  | ec:3.1.3.11 | fructose-bisphosphatase | 5 |
|  | ec:4.1.2.13 | fructose-bisphosphate aldolase | 4 |
|  | ec:1.1.1.49 | glucose-6-phosphate dehydrogenase | 6 |
|  | ec:1.1.1.44 | phosphogluconate dehydrogenase (decarboxylating) | 7 |
|  | ec:5.4.2.2 | phosphoglucomutase | 3 |
| **Starch and sucrose metabolism** | ec:4.1.1.35 | UDP-glucuronate decarboxylase | 8 |
|  | ec:2.4.1.1 | phosphorylase | 3 |
|  | ec:5.3.1.9 | glucose-6-phosphate isomerase | 3 |
|  | ec:2.7.1.4 | fructokinase | 3 |
|  | ec:2.7.1.2 | glucokinase | 1 |
|  | ec:2.7.1.1 | hexokinase | 1 |
|  | ec:3.2.1.4 | cellulase | 10 |
|  | ec:3.2.1.2 | beta-amylase | 4 |
|  | ec:3.2.1.1 | alpha-amylase | 5 |
|  | ec:3.2.1.67 | galacturan 1,4-alpha-galacturonidase | 1 |
|  | ec:3.1.1.11 | pectinesterase | 28 |
|  | ec:3.2.1.39 | glucan endo-1,3-beta-D-glucosidase | 12 |
|  | ec:3.2.1.28 | alpha,alpha-trehalase | 2 |
|  | ec:3.2.1.26 | beta-fructofuranosidase | 3 |
|  | ec:3.2.1.21 | beta-glucosidase | 7 |
|  | ec:3.2.1.20 | alpha-glucosidase | 6 |
|  | ec:3.2.1.15 | polygalacturonase | 12 |
|  | ec:2.7.7.9 | UTP---glucose-1-phosphate uridylyltransferase | 1 |
|  | ec:3.1.3.24 | sucrose-phosphate phosphatase | 3 |
|  | ec:3.1.3.12 | trehalose-phosphatase | 7 |
|  | ec:2.7.7.27 | glucose-1-phosphate adenylyltransferase | 3 |
|  | ec:2.4.1.34 | 1,3-beta-glucan synthase | 8 |
|  | ec:3.6.1.21 | ADP-sugar diphosphatase | 1 |
|  | ec:2.4.1.25 | 4-alpha-glucanotransferase | 3 |
|  | ec:2.4.1.21 | starch synthase | 11 |
|  | ec:2.4.1.18 | 1,4-alpha-glucan branching enzyme | 2 |
|  | ec:2.4.1.15 | alpha,alpha-trehalose-phosphate synthase (UDP-forming) | 6 |
|  | ec:2.4.1.14 | sucrose-phosphate synthase | 2 |
|  | ec:2.4.1.13 | sucrose synthase | 1 |
|  | ec:2.4.1.12 | cellulose synthase (UDP-forming) | 16 |
|  | ec:1.1.1.22 | UDP-glucose 6-dehydrogenase | 3 |
|  | ec:5.4.2.6 | beta-phosphoglucomutase | 1 |
|  | ec:5.4.2.2 | phosphoglucomutase | 3 |
| **Glycolysis / Gluconeogenesis** | ec:2.7.1.11 | 6-phosphofructokinase | 10 |
|  | ec:4.1.1.49 | phosphoenolpyruvate carboxykinase (ATP) | 1 |
|  | ec:5.3.1.9 | glucose-6-phosphate isomerase | 3 |
|  | ec:5.3.1.1 | triose-phosphate isomerase | 3 |
|  | ec:2.7.1.2 | glucokinase | 1 |
|  | ec:2.7.1.1 | hexokinase | 1 |
|  | ec:4.2.1.11 | phosphopyruvate hydratase | 5 |
|  | ec:5.1.3.3 | aldose 1-epimerase | 2 |
|  | ec:1.1.1.1 | alcohol dehydrogenase | 4 |
|  | ec:3.1.3.11 | fructose-bisphosphatase | 5 |
|  | ec:6.2.1.1 | acetate---CoA ligase | 2 |
|  | ec:2.7.2.3 | phosphoglycerate kinase | 2 |
|  | ec:4.1.1.1 | pyruvate decarboxylase | 1 |
|  | ec:1.2.1.9 | glyceraldehyde-3-phosphate dehydrogenase (NADP+) | 1 |
|  | ec:1.2.1.3 | aldehyde dehydrogenase (NAD+) | 4 |
|  | ec:4.1.2.13 | fructose-bisphosphate aldolase | 4 |
|  | ec:1.1.1.27 | L-lactate dehydrogenase | 1 |
|  | ec:1.2.4.1 | pyruvate dehydrogenase (acetyl-transferring) | 4 |
|  | ec:1.8.1.4 | dihydrolipoyl dehydrogenase | 2 |
|  | ec:1.2.1.12 | glyceraldehyde-3-phosphate dehydrogenase (phosphorylating) | 7 |
|  | ec:5.4.2.2 | phosphoglucomutase | 3 |
|  | ec:5.4.2.1 | phosphoglycerate mutase | 2 |
|  | ec:2.7.1.40 | pyruvate kinase | 4 |
| **Citrate cycle (TCA cycle)** | ec:4.1.1.49 | phosphoenolpyruvate carboxykinase (ATP) | 1 |
|  | ec:2.3.3.8 | ATP citrate synthase | 6 |
|  | ec:2.3.3.1 | citrate (Si)-synthase | 1 |
|  | ec:6.2.1.5 | succinate---CoA ligase (ADP-forming) | 7 |
|  | ec:2.3.1.61 | dihydrolipoyllysine-residue succinyltransferase | 1 |
|  | ec:1.3.5.1 | succinate dehydrogenase (ubiquinone) | 1 |
|  | ec:1.1.1.42 | isocitrate dehydrogenase (NADP+) | 4 |
|  | ec:1.1.1.41 | isocitrate dehydrogenase (NAD+) | 3 |
|  | ec:1.1.1.37 | malate dehydrogenase | 7 |
|  | ec:1.2.4.2 | oxoglutarate dehydrogenase (succinyl-transferring) | 4 |
|  | ec:1.2.4.1 | pyruvate dehydrogenase (acetyl-transferring) | 4 |
|  | ec:1.8.1.4 | dihydrolipoyl dehydrogenase | 2 |
|  | ec:4.2.1.3 | aconitate hydratase | 2 |
|  | ec:4.2.1.2 | fumarate hydratase | 3 |
| **Pyruvate metabolism** | ec:4.1.1.49 | phosphoenolpyruvate carboxykinase (ATP) | 1 |
|  | ec:4.1.1.31 | phosphoenolpyruvate carboxylase | 7 |
|  | ec:6.4.1.2 | acetyl-CoA carboxylase | 7 |
|  | ec:3.1.2.6 | hydroxyacylglutathione hydrolase | 2 |
|  | ec:2.3.3.9 | malate synthase | 1 |
|  | ec:6.2.1.1 | acetate---CoA ligase | 2 |
|  | ec:1.1.1.82 | malate dehydrogenase (NADP+) | 2 |
|  | ec:4.4.1.5 | lactoylglutathione lyase | 3 |
|  | ec:1.2.1.3 | aldehyde dehydrogenase (NAD+) | 4 |
|  | ec:1.1.1.39 | malate dehydrogenase (decarboxylating) | 2 |
|  | ec:1.1.1.37 | malate dehydrogenase | 7 |
|  | ec:1.1.1.27 | L-lactate dehydrogenase | 1 |
|  | ec:1.1.1.21 | aldehyde reductase | 1 |
|  | ec:1.2.4.1 | pyruvate dehydrogenase (acetyl-transferring) | 4 |
|  | ec:2.3.3.13 | 2-isopropylmalate synthase | 4 |
|  | ec:1.8.1.4 | dihydrolipoyl dehydrogenase | 2 |
|  | ec:2.7.1.40 | pyruvate kinase | 4 |
| **Oxidative phosphorylation** | ec:1.10.2.2 | ubiquinol---cytochrome-c reductase | 5 |
|  | ec:3.6.3.6 | H+-exporting ATPase | 8 |
|  | ec:1.3.5.1 | succinate dehydrogenase (ubiquinone) | 1 |
|  | ec:1.6.5.3 | NADH:ubiquinone reductase (H+-translocating) | 13 |
|  | ec:3.6.1.1 | inorganic diphosphatase ec:3.6.1.10 endopolyphosphatase | 9 |
|  | ec:1.9.3.1 | cytochrome-c oxidase | 7 |
| **Pantothenate and CoA biosynthesis** | ec:4.1.1.36 | phosphopantothenoylcysteine decarboxylase | 2 |
|  | ec:2.2.1.6 | acetolactate synthase | 3 |
|  | ec:1.1.1.86 | ketol-acid reductoisomerase | 2 |
|  | ec:2.7.8.7 | holo-[acyl-carrier-protein] synthase | 1 |
|  | ec:2.6.1.42 | branched-chain-amino-acid transaminase | 3 |
|  | ec:2.7.1.33 | pantothenate kinase | 1 |
|  | ec:4.2.1.9 | dihydroxy-acid dehydratase | 1 |
| **Folate biosynthesis** | ec:2.7.6.3 | 2-amino-4-hydroxy-6-hydroxymethyldihydropteridine diphosphokinase | 1 |
|  | ec:3.5.4.16 | GTP cyclohydrolase I | 1 |
|  | ec:4.1.2.25 | dihydroneopterin aldolase | 1 |
|  | ec:1.5.1.3 | dihydrofolate reductase | 1 |
|  | ec:2.5.1.15 | dihydropteroate synthase | 1 |
| **One carbon pool by folate** | ec:6.3.4.3 | formate---tetrahydrofolate ligase | 1 |
|  | ec:2.1.2.10 | aminomethyltransferase | 1 |
|  | ec:1.5.1.20 | methylenetetrahydrofolate reductase [NAD(P)H] | 1 |
|  | ec:2.1.1.45 | thymidylate synthase | 1 |
|  | ec:2.1.2.3 | phosphoribosylaminoimidazolecarboxamide formyltransferase | 2 |
|  | ec:2.1.2.2 | phosphoribosylglycinamide formyltransferase | 1 |
|  | ec:2.1.2.1 | glycine hydroxymethyltransferase | 6 |
|  | ec:3.5.4.9 | methenyltetrahydrofolate cyclohydrolase | 2 |
|  | ec:6.3.3.2 | 5-formyltetrahydrofolate cyclo-ligase | 1 |
|  | ec:1.5.1.5 | methylenetetrahydrofolate dehydrogenase (NADP+) | 2 |
|  | ec:1.5.1.3 | dihydrofolate reductase | 1 |
| **Fatty acid biosynthesis** | ec:1.3.1.9 | enoyl-[acyl-carrier-protein] reductase (NADH) | 2 |
|  | ec:6.4.1.2 | acetyl-CoA carboxylase | 7 |
|  | ec:1.1.1.100 | 3-oxoacyl-[acyl-carrier-protein] reductase | 7 |
|  | ec:2.3.1.41 | beta-ketoacyl-acyl-carrier-protein synthase I | 6 |
|  | ec:2.3.1.39 | [acyl-carrier-protein] S-malonyltransferase | 1 |
|  | ec:1.14.19.2 | acyl-[acyl-carrier-protein] desaturase | 4 |
| **Biosynthesis of unsaturated fatty acids** | ec:4.2.1.17 | enoyl-CoA hydratase | 2 |
|  | ec:1.1.1.100 | 3-oxoacyl-[acyl-carrier-protein] reductase | 7 |
|  | ec:1.14.19.2 | acyl-[acyl-carrier-protein] desaturase | 4 |
|  | ec:1.3.3.6 | acyl-CoA oxidase | 4 |
| **alpha-Linolenic acid metabolism** | ec:5.3.99.6 | allene-oxide cyclase | 1 |
|  | ec:4.2.1.92 | hydroperoxide dehydratase | 2 |
|  | ec:2.1.1.141 | jasmonate O-methyltransferase | 1 |
|  | ec:4.2.1.17 | enoyl-CoA hydratase | 2 |
|  | ec:1.13.11.12 | linoleate 13S-lipoxygenase | 9 |
|  | ec:1.3.3.6 | acyl-CoA oxidase | 4 |
|  | ec:3.1.1.4 | phospholipase A2 | 4 |
|  | ec:1.3.1.42 | 12-oxophytodienoate reductase | 2 |
| **Glycerolipid metabolism** | ec:3.2.1.22 | alpha-galactosidase | 4 |
|  | ec:2.3.1.15 | glycerol-3-phosphate O-acyltransferase | 1 |
|  | ec:2.4.1.46 | monogalactosyldiacylglycerol synthase | 2 |
|  | ec:1.2.1.3 | aldehyde dehydrogenase (NAD+) | 4 |
|  | ec:2.7.1.107 | diacylglycerol kinase | 8 |
|  | ec:1.1.1.21 | aldehyde reductase | 1 |
|  | ec:2.4.1.241 | digalactosyldiacylglycerol synthase | 1 |
|  | ec:3.1.1.3 | triacylglycerol lipase | 23 |
|  | ec:2.7.1.29 | glycerone kinase | 1 |
| **Glycerophospholipid metabolism** | ec:3.1.4.46 | glycerophosphodiester phosphodiesterase | 2 |
|  | ec:4.1.1.65 | phosphatidylserine decarboxylase | 2 |
|  | ec:3.1.4.4 | phospholipase D | 9 |
|  | ec:3.1.4.3 | phospholipase C | 1 |
|  | ec:2.7.8.11 | CDP-diacylglycerol---inositol 3-phosphatidyltransferase | 1 |
|  | ec:1.1.1.8 | glycerol-3-phosphate dehydrogenase (NAD+) | 1 |
|  | ec:2.7.7.41 | phosphatidate cytidylyltransferase | 3 |
|  | ec:2.3.1.43 | phosphatidylcholine---sterol O-acyltransferase | 6 |
|  | ec:2.3.1.15 | glycerol-3-phosphate O-acyltransferase | 1 |
|  | ec:2.7.1.107 | diacylglycerol kinase | 8 |
|  | ec:2.7.8.5 | CDP-diacylglycerol---glycerol-3-phosphate 3-phosphatidyltransferase | 1 |
|  | ec:3.1.1.4 | phospholipase A2 | 4 |
| **Sphingolipid metabolism** | ec:3.2.1.45 | glucosylceramidase | 2 |
|  | ec:3.2.1.23 | beta-galactosidase | 20 |
|  | ec:3.2.1.22 | alpha-galactosidase | 4 |
|  | ec:1.1.1.102 | 3-dehydrosphinganine reductase | 1 |
|  | ec:2.3.1.50 | serine C-palmitoyltransferase | 2 |
|  | ec:3.5.1.23 | ceramidase | 1 |
|  | ec:2.7.1.138 | ceramide kinase | 1 |
| **Carotenoid biosynthesis** | ec:1.10.99.3 | violaxanthin de-epoxidase | 1 |
|  | ec:1.14.13.90 | zeaxanthin epoxidase | 1 |
|  | ec:2.5.1.32 | phytoene synthase | 2 |
| **Porphyrin and chlorophyll metabolism** | ec:4.1.1.37 | uroporphyrinogen decarboxylase | 1 |
|  | ec:4.99.1.3 | sirohydrochlorin cobaltochelatase | 1 |
|  | ec:4.99.1.1 | ferrochelatase | 1 |
|  | ec:5.4.3.8 | glutamate-1-semialdehyde 2,1-aminomutase | 2 |
|  | ec:1.3.7.4 | phytochromobilin:ferredoxin oxidoreductase | 1 |
|  | ec:1.14.99.3 | heme oxygenase | 2 |
|  | ec:2.1.1.11 | magnesium protoporphyrin IX methyltransferase ec:2.1.1.110 sterigmatocystin 8-O-methyltransferase | 1 |
|  | ec:1.14.13.81 | magnesium-protoporphyrin IX monomethyl ester (oxidative) cyclase | 1 |
|  | ec:2.5.1.61 | hydroxymethylbilane synthase | 1 |
|  | ec:6.1.1.17 | glutamate---tRNA ligase | 1 |
|  | ec:1.2.1.70 | glutamyl-tRNA reductase | 1 |
|  | ec:1.3.3.5 | bilirubin oxidase | 1 |
|  | ec:1.3.3.4 | protoporphyrinogen oxidase | 2 |
|  | ec:1.3.3.3 | coproporphyrinogen oxidase | 1 |
|  | ec:1.3.1.33 | protochlorophyllide reductase | 5 |
|  | ec:6.6.1.1 | magnesium chelatase | 4 |
| **Nitrogen metabolism** | ec:2.1.2.10 | aminomethyltransferase | 1 |
|  | ec:4.2.1.104 | cyanase | 1 |
|  | ec:3.5.1.1 | asparaginase | 1 |
|  | ec:6.3.5.4 | asparagine synthase (glutamine-hydrolysing) | 2 |
|  | ec:1.13.12.16 | nitronate monooxygenase | 1 |
|  | ec:1.4.1.2 | glutamate dehydrogenase | 1 |
|  | ec:1.7.1.1 | nitrate reductase (NADH) | 2 |
|  | ec:4.4.1.8 | cystathionine beta-lyase | 1 |
|  | ec:1.7.7.1 | ferredoxin---nitrite reductase | 1 |
|  | ec:6.3.1.2 | glutamate---ammonia ligase | 4 |
|  | ec:4.2.1.1 | carbonate dehydratase | 5 |
| **Alanine, glutamate and aspartate metabolism** | ec:4.1.1.15 | glutamate decarboxylase | 4 |
|  | ec:4.2.1.17 | enoyl-CoA hydratase | 2 |
|  | ec:1.4.3.21 | primary-amine oxidase | 4 |
|  | ec:1.2.1.3 | aldehyde dehydrogenase (NAD+) | 4 |
|  | ec:2.5.1.16 | spermidine synthase | 2 |
|  | ec:1.2.1.19 | aminobutyraldehyde dehydrogenase | 1 |
|  | ec:1.3.99.3 | acyl-CoA dehydrogenase | 6 |
|  | ec:2.6.1.19 | 4-aminobutyrate transaminase | 6 |
|  | ec:6.3.4.4 | adenylosuccinate synthase | 4 |
|  | ec:4.1.1.15 | glutamate decarboxylase | 4 |
|  | ec:1.4.3.2 | L-amino-acid oxidase | 1 |
|  | ec:1.4.3.16 | L-aspartate oxidase | 1 |
|  | ec:2.4.2.14 | amidophosphoribosyltransferase | 1 |
|  | ec:3.5.1.1 | asparaginase | 1 |
|  | ec:6.3.5.4 | asparagine synthase (glutamine-hydrolysing) | 2 |
|  | ec:1.4.1.2 | glutamate dehydrogenase | 1 |
|  | ec:4.3.2.2 | adenylosuccinate lyase | 1 |
|  | ec:2.1.3.2 | aspartate carbamoyltransferase | 1 |
|  | ec:2.6.1.1 | aspartate transaminase | 1 |
|  | ec:6.3.1.2 | glutamate---ammonia ligase | 4 |
|  | ec:2.6.1.19 | 4-aminobutyrate transaminase | 6 |
|  | ec:2.6.1.16 | glutamine---fructose-6-phosphate transaminase (isomerizing) | 1 |
| **Glycine, serine and threonine metabolism** | ec:2.1.2.10 | aminomethyltransferase | 1 |
|  | ec:4.2.1.20 | tryptophan synthase | 5 |
|  | ec:1.4.3.21 | primary-amine oxidase | 4 |
|  | ec:4.3.1.19 | threonine ammonia-lyase | 2 |
|  | ec:1.1.1.3 | homoserine dehydrogenase | 3 |
|  | ec:1.1.1.1 | alcohol dehydrogenase | 4 |
|  | ec:2.1.2.1 | glycine hydroxymethyltransferase | 6 |
|  | ec:1.4.4.2 | glycine dehydrogenase (decarboxylating) | 1 |
|  | ec:2.7.2.4 | aspartate kinase | 2 |
|  | ec:1.1.1.95 | phosphoglycerate dehydrogenase | 2 |
|  | ec:4.2.3.1 | threonine synthase | 2 |
|  | ec:1.1.1.29 | glycerate dehydrogenase | 1 |
|  | ec:1.8.1.4 | dihydrolipoyl dehydrogenase | 2 |
| **Lysine biosynthesis** | ec:4.2.1.52 | dihydrodipicolinate synthase | 3 |
|  | ec:1.1.1.3 | homoserine dehydrogenase | 3 |
|  | ec:2.7.2.4 | aspartate kinase | 2 |
|  | ec:1.3.1.26 | dihydrodipicolinate reductase | 2 |
| **Valine, leucine and isoleucine degradation** | ec:1.4.3.2 | L-amino-acid oxidase | 1 |
|  | ec:4.2.1.18 | methylglutaconyl-CoA hydratase | 1 |
|  | ec:4.2.1.17 | enoyl-CoA hydratase | 2 |
|  | ec:1.3.99.10 | isovaleryl-CoA dehydrogenase | 1 |
|  | ec:4.1.3.4 | hydroxymethylglutaryl-CoA lyase | 2 |
|  | ec:1.2.3.1 | aldehyde oxidase | 5 |
|  | ec:2.3.1.168 | dihydrolipoyllysine-residue (2-methylpropanoyl)transferase | 1 |
|  | ec:1.2.1.3 | aldehyde dehydrogenase (NAD+) | 4 |
|  | ec:1.1.1.35 | 3-hydroxyacyl-CoA dehydrogenase | 1 |
|  | ec:1.1.1.31 | 3-hydroxyisobutyrate dehydrogenase | 2 |
|  | ec:2.3.3.10 | hydroxymethylglutaryl-CoA synthase | 6 |
|  | ec:1.8.1.4 | dihydrolipoyl dehydrogenase | 2 |
|  | ec:2.6.1.42 | branched-chain-amino-acid transaminase | 3 |
|  | ec:1.2.1.27 | methylmalonate-semialdehyde dehydrogenase (acylating) | 1 |
|  | ec:1.3.99.3 | acyl-CoA dehydrogenase | 6 |
| **Methionine and cysteine metabolism** | ec:4.1.1.50 | adenosylmethionine decarboxylase | 3 |
|  | ec:1.4.3.2 | L-amino-acid oxidase | 1 |
|  | ec:1.1.1.3 | homoserine dehydrogenase | 3 |
|  | ec:4.4.1.14 | 1-aminocyclopropane-1-carboxylate synthase | 13 |
|  | ec:2.1.1.37 | DNA (cytosine-5-)-methyltransferase | 4 |
|  | ec:2.1.1.14 | 5-methyltetrahydropteroyltriglutamate---homocysteine S-methyltransferase | 1 |
|  | ec:2.5.1.6 | methionine adenosyltransferase | 5 |
|  | ec:2.1.1.10 | homocysteine S-methyltransferase | 1 |
|  | ec:2.7.2.4 | aspartate kinase | 2 |
|  | ec:2.3.1.30 | serine O-acetyltransferase | 4 |
|  | ec:3.3.1.1 | adenosylhomocysteinase | 6 |
|  | ec:4.4.1.8 | cystathionine beta-lyase | 1 |
|  | ec:2.5.1.47 | cysteine synthase | 8 |
|  | ec:2.7.1.100 | S-methyl-5-thioribose kinase | 1 |
|  | ec:1.1.1.27 | L-lactate dehydrogenase | 1 |
|  | ec:2.5.1.16 | spermidine synthase | 2 |
|  | ec:2.6.1.1 | aspartate transaminase | 1 |
|  | ec:1.14.17.4 | aminocyclopropanecarboxylate oxidase | 4 |
| **Arginine and proline metabolism** | ec:4.1.1.50 | adenosylmethionine decarboxylase | 3 |
|  | ec:1.5.99.8 | proline dehydrogenase | 3 |
|  | ec:3.5.3.1 | arginase | 2 |
|  | ec:4.1.1.19 | arginine decarboxylase | 2 |
|  | ec:4.1.1.17 | ornithine decarboxylase | 1 |
|  | ec:3.5.1.5 | urease ec:3.5.1.50 pentanamidase | 1 |
|  | ec:3.5.1.4 | amidase | 4 |
|  | ec:1.4.1.2 | glutamate dehydrogenase | 1 |
|  | ec:3.5.1.53 | N-carbamoylputrescine amidase | 2 |
|  | ec:2.7.2.11 | glutamate 5-kinase | 3 |
|  | ec:1.2.1.3 | aldehyde dehydrogenase (NAD+) | 4 |
|  | ec:2.3.1.1 | amino-acid N-acetyltransferase | 1 |
|  | ec:2.5.1.16 | spermidine synthase | 2 |
|  | ec:1.2.1.41 | glutamate-5-semialdehyde dehydrogenase | 2 |
|  | ec:3.5.3.12 | agmatine deiminase | 1 |
|  | ec:2.6.1.1 | aspartate transaminase | 1 |
|  | ec:1.2.1.38 | N-acetyl-gamma-glutamyl-phosphate reductase | 1 |
|  | ec:1.2.1.19 | aminobutyraldehyde dehydrogenase | 1 |
|  | ec:6.3.1.2 | glutamate---ammonia ligase | 4 |
| **Ascorbate and aldarate metabolism** | ec:5.1.3.18 | GDP-mannose 3,5-epimerase | 2 |
|  | ec:1.11.1.11 | L-ascorbate peroxidase | 6 |
|  | ec:1.8.5.1 | glutathione dehydrogenase (ascorbate) | 2 |
|  | ec:1.13.99.1 | inositol oxygenase | 4 |
|  | ec:1.3.2.3 | L-galactonolactone dehydrogenase | 1 |
|  | ec:1.6.5.4 | monodehydroascorbate reductase (NADH) | 3 |
|  | ec:1.10.3.3 | L-ascorbate oxidase | 6 |
|  | ec:1.2.1.3 | aldehyde dehydrogenase (NAD+) | 4 |
|  | ec:1.1.1.22 | UDP-glucose 6-dehydrogenase | 3 |
| **Tryptophan metabolism** | ec:4.1.1.28 | aromatic-L-amino-acid decarboxylase | 4 |
|  | ec:1.4.3.2 | L-amino-acid oxidase | 1 |
|  | ec:4.2.1.17 | enoyl-CoA hydratase | 2 |
|  | ec:1.11.1.6 | catalase | 3 |
|  | ec:3.5.1.4 | amidase | 4 |
|  | ec:1.2.3.1 | aldehyde oxidase | 5 |
|  | ec:1.2.1.3 | aldehyde dehydrogenase (NAD+) | 4 |
|  | ec:1.1.1.35 | 3-hydroxyacyl-CoA dehydrogenase | 1 |
|  | ec:1.2.4.2 | oxoglutarate dehydrogenase (succinyl-transferring) | 4 |
|  | ec:1.14.14.1 | unspecific monooxygenase | 1 |
| **Histidine metabolism** | ec:4.1.1.28 | aromatic-L-amino-acid decarboxylase | 4 |
|  | ec:4.1.1.22 | histidine decarboxylase | 3 |
|  | ec:4.2.1.19 | imidazoleglycerol-phosphate dehydratase | 1 |
|  | ec:1.2.1.3 | aldehyde dehydrogenase (NAD+) | 4 |
|  | ec:1.1.1.23 | histidinol dehydrogenase | 1 |
|  | ec:2.6.1.9 | histidinol-phosphate transaminase | 1 |
| **Phenylalanine, tyrosine and tryptophan biosynthesis** | ec:4.2.1.91 | arogenate dehydratase | 1 |
|  | ec:5.4.99.5 | chorismate mutase | 2 |
|  | ec:4.2.1.51 | prephenate dehydratase | 5 |
|  | ec:1.4.3.2 | L-amino-acid oxidase | 1 |
|  | ec:4.2.1.20 | tryptophan synthase | 5 |
|  | ec:4.2.1.10 | 3-dehydroquinate dehydratase | 3 |
|  | ec:4.1.3.27 | anthranilate synthase | 4 |
|  | ec:2.4.2.18 | anthranilate phosphoribosyltransferase | 1 |
|  | ec:4.2.3.5 | chorismate synthase | 3 |
|  | ec:4.2.3.4 | 3-dehydroquinate synthase | 1 |
|  | ec:2.5.1.54 | 3-deoxy-7-phosphoheptulonate synthase | 3 |
|  | ec:1.1.1.25 | shikimate dehydrogenase | 4 |
|  | ec:2.5.1.19 | 3-phosphoshikimate 1-carboxyvinyltransferase | 3 |
|  | ec:2.6.1.9 | histidinol-phosphate transaminase | 1 |
|  | ec:2.6.1.1 | aspartate transaminase | 1 |
|  | ec:2.7.1.71 | shikimate kinase | 2 |
|  | ec:1.3.1.13 | prephenate dehydrogenase (NADP+) | 1 |
| **Tyrosine metabolism** | ec:4.1.1.68 | 5-oxopent-3-ene-1,2,5-tricarboxylate decarboxylase | 1 |
|  | ec:2.1.1.6 | catechol O-methyltransferase | 1 |
|  | ec:3.7.1.2 | fumarylacetoacetase | 3 |
|  | ec:4.1.1.28 | aromatic-L-amino-acid decarboxylase | 4 |
|  | ec:1.4.3.2 | L-amino-acid oxidase | 1 |
|  | ec:1.4.3.21 | primary-amine oxidase | 4 |
|  | ec:1.1.1.1 | alcohol dehydrogenase | 4 |
|  | ec:1.2.3.1 | aldehyde oxidase | 5 |
|  | ec:1.13.11.5 | homogentisate 1,2-dioxygenase | 1 |
|  | ec:1.10.3.1 | catechol oxidase | 8 |
|  | ec:5.2.1.2 | maleylacetoacetate isomerase | 1 |
|  | ec:2.6.1.9 | histidinol-phosphate transaminase | 1 |
|  | ec:2.6.1.1 | aspartate transaminase | 1 |
| **Phenylalanine metabolism** | ec:4.1.1.28 | aromatic-L-amino-acid decarboxylase | 4 |
|  | ec:1.4.3.2 | L-amino-acid oxidase | 1 |
|  | ec:1.4.3.21 | primary-amine oxidase | 4 |
|  | ec:2.1.1.104 | caffeoyl-CoA O-methyltransferase | 4 |
|  | ec:1.11.1.7 | peroxidase | 56 |
|  | ec:3.5.1.4 | amidase | 4 |
|  | ec:2.6.1.9 | histidinol-phosphate transaminase | 1 |
|  | ec:2.6.1.1 | aspartate transaminase | 1 |
|  | ec:1.14.13.11 | trans-cinnamate 4-monooxygenase | 1 |
|  | ec:6.2.1.12 | 4-coumarate---CoA ligase | 4 |
| **N-Glycan biosynthesis** | ec:2.4.2.38 | glycoprotein 2-beta-D-xylosyltransferase | 1 |
|  | ec:3.2.1.52 | beta-N-acetylhexosaminidase | 2 |
|  | ec:2.4.1.119 | dolichyl-diphosphooligosaccharide---protein glycotransferase | 1 |
|  | ec:2.4.1.101 | alpha-1,3-mannosyl-glycoprotein 2-beta-N-acetylglucosaminyltransferase | 2 |
|  | ec:3.2.1.113 | mannosyl-oligosaccharide 1,2-alpha-mannosidase | 1 |
| **Purine metabolism** | ec:6.3.4.4 | adenylosuccinate synthase | 4 |
|  | ec:2.7.6.1 | ribose-phosphate diphosphokinase | 1 |
|  | ec:4.1.1.21 | phosphoribosylaminoimidazole carboxylase | 1 |
|  | ec:1.7.3.3 | factor-independent urate hydroxylase | 1 |
|  | ec:6.3.2.6 | phosphoribosylaminoimidazolesuccinocarboxamide synthase | 2 |
|  | ec:2.7.4.8 | guanylate kinase | 2 |
|  | ec:2.7.4.6 | nucleoside-diphosphate kinase | 2 |
|  | ec:2.7.4.3 | adenylate kinase | 6 |
|  | ec:2.4.2.14 | amidophosphoribosyltransferase | 1 |
|  | ec:3.5.1.5 | urease ec:3.5.1.50 pentanamidase | 1 |
|  | ec:6.3.5.3 | phosphoribosylformylglycinamidine synthase | 2 |
|  | ec:6.3.5.2 | GMP synthase (glutamine-hydrolysing) | 1 |
|  | ec:4.6.1.1 | adenylate cyclase | 4 |
|  | ec:2.1.2.3 | phosphoribosylaminoimidazolecarboxamide formyltransferase | 2 |
|  | ec:2.1.2.2 | phosphoribosylglycinamide formyltransferase | 1 |
|  | ec:3.5.4.10 | IMP cyclohydrolase | 2 |
|  | ec:2.7.7.8 | polyribonucleotide nucleotidyltransferase | 1 |
|  | ec:2.7.7.7 | DNA-directed DNA polymerase | 7 |
|  | ec:2.7.7.6 | DNA-directed RNA polymerase | 25 |
|  | ec:2.7.7.4 | sulfate adenylyltransferase | 5 |
|  | ec:3.5.4.6 | AMP deaminase | 1 |
|  | ec:3.5.4.4 | adenosine deaminase | 2 |
|  | ec:2.7.7.48 | RNA-directed RNA polymerase | 6 |
|  | ec:2.4.2.8 | hypoxanthine phosphoribosyltransferase | 1 |
|  | ec:2.4.2.7 | adenine phosphoribosyltransferase | 1 |
|  | ec:1.17.4.1 | ribonucleoside-diphosphate reductase | 4 |
|  | ec:6.3.3.1 | phosphoribosylformylglycinamidine cyclo-ligase | 1 |
|  | ec:3.1.3.5 | 5'-nucleotidase | 3 |
|  | ec:3.6.1.3 | adenosinetriphosphatase | 26 |
|  | ec:4.3.2.2 | adenylosuccinate lyase | 1 |
|  | ec:3.6.1.21 | ADP-sugar diphosphatase | 1 |
|  | ec:6.3.4.13 | phosphoribosylamine---glycine ligase | 1 |
|  | ec:3.6.1.15 | nucleoside-triphosphatase | 53 |
|  | ec:3.6.1.11 | exopolyphosphatase | 1 |
|  | ec:1.1.1.205 | IMP dehydrogenase | 1 |
|  | ec:5.4.2.2 | phosphoglucomutase | 3 |
|  | ec:2.7.1.40 | pyruvate kinase | 4 |
|  | ec:2.7.1.25 | adenylyl-sulfate kinase | 2 |
|  | ec:2.7.1.20 | adenosine kinase | 2 |
| **Pyrimidine metabolism** | ec:6.3.4.2 | CTP synthase | 1 |
|  | ec:4.1.1.23 | orotidine-5'-phosphate decarboxylase | 1 |
|  | ec:2.7.4.6 | nucleoside-diphosphate kinase | 2 |
|  | ec:2.4.2.10 | orotate phosphoribosyltransferase | 1 |
|  | ec:2.1.1.45 | thymidylate synthase | 1 |
|  | ec:2.7.7.8 | polyribonucleotide nucleotidyltransferase | 1 |
|  | ec:2.7.7.7 | DNA-directed DNA polymerase | 7 |
|  | ec:2.7.7.6 | DNA-directed RNA polymerase | 25 |
|  | ec:2.4.2.9 | uracil phosphoribosyltransferase | 1 |
|  | ec:1.17.4.1 | ribonucleoside-diphosphate reductase | 4 |
|  | ec:3.1.3.5 | 5'-nucleotidase | 3 |
|  | ec:3.6.1.23 | dUTP diphosphatase | 2 |
|  | ec:2.1.3.2 | aspartate carbamoyltransferase | 1 |
|  | ec:2.7.1.48 | uridine kinase | 3 |
|  | ec:2.7.1.21 | thymidine kinase | 2 |
| **Nicotinate and nicotinamide metabolism** | ec:1.4.3.16 | L-aspartate oxidase | 1 |
|  | ec:2.4.2.19 | nicotinate-nucleotide diphosphorylase (carboxylating) | 1 |
|  | ec:2.4.2.11 | nicotinate phosphoribosyltransferase | 1 |
|  | ec:6.3.5.1 | NAD+ synthase (glutamine-hydrolysing) | 1 |
|  | ec:1.2.3.1 | aldehyde oxidase | 5 |
|  | ec:3.1.3.5 | 5'-nucleotidase | 3 |
|  | ec:3.6.1.22 | NAD+ diphosphatase | 1 |
|  | ec:2.7.1.23 | NAD+ kinase | 1 |
| **Inositol phosphate metabolism** | ec:3.1.4.3 | phospholipase C | 1 |
|  | ec:3.1.4.11 | phosphoinositide phospholipase C | 8 |
|  | ec:2.7.8.11 | CDP-diacylglycerol---inositol 3-phosphatidyltransferase | 1 |
|  | ec:5.3.1.1 | triose-phosphate isomerase | 3 |
|  | ec:1.13.99.1 | inositol oxygenase | 4 |
|  | ec:3.1.3.57 | inositol-1,4-bisphosphate 1-phosphatase | 1 |
|  | ec:3.1.3.25 | inositol-phosphate phosphatase | 1 |
|  | ec:2.7.1.159 | inositol-1,3,4-trisphosphate 5/6-kinase | 1 |
|  | ec:2.7.1.150 | 1-phosphatidylinositol-3-phosphate 5-kinase | 2 |
|  | ec:2.7.1.137 | phosphatidylinositol 3-kinase | 2 |
|  | ec:2.7.1.134 | inositol-tetrakisphosphate 1-kinase | 1 |
|  | ec:2.7.1.127 | inositol-trisphosphate 3-kinase | 2 |
|  | ec:5.5.1.4 | inositol-3-phosphate synthase | 3 |
|  | ec:2.7.1.68 | 1-phosphatidylinositol-4-phosphate 5-kinase | 3 |
|  | ec:2.7.1.67 | 1-phosphatidylinositol 4-kinase | 3 |
|  | ec:4.1.1.28 | aromatic-L-amino-acid decarboxylase | 4 |
| **Riboflavin metabolism** | ec:1.1.1.193 | 5-amino-6-(5-phosphoribosylamino)uracil reductase | 1 |
|  | ec:3.5.4.26 | diaminohydroxyphosphoribosylaminopyrimidine deaminase | 1 |
|  | ec:3.5.4.25 | GTP cyclohydrolase II | 6 |
|  | ec:2.5.1.9 | riboflavin synthase | 3 |
|  | ec:3.1.3.2 | acid phosphatase | 10 |
|  | ec:4.1.99.12 | 3,4-dihydroxy-2-butanone-4-phosphate synthase | 6 |
|  | ec:2.7.1.26 | riboflavin kinase | 1 |
| **Glutathione metabolism** | ec:4.1.1.17 | ornithine decarboxylase | 1 |
|  | ec:2.3.2.2 | gamma-glutamyltransferase | 1 |
|  | ec:6.3.2.2 | glutamate---cysteine ligase | 1 |
|  | ec:1.11.1.9 | glutathione peroxidase | 4 |
|  | ec:1.11.1.15 | peroxiredoxin | 1 |
|  | ec:1.11.1.12 | phospholipid-hydroperoxide glutathione peroxidase | 1 |
|  | ec:1.11.1.11 | L-ascorbate peroxidase | 6 |
|  | ec:1.8.5.1 | glutathione dehydrogenase (ascorbate) | 2 |
|  | ec:1.17.4.1 | ribonucleoside-diphosphate reductase | 4 |
|  | ec:1.1.1.49 | glucose-6-phosphate dehydrogenase | 6 |
|  | ec:1.1.1.44 | phosphogluconate dehydrogenase (decarboxylating) | 7 |
|  | ec:1.1.1.42 | isocitrate dehydrogenase (NADP+) | 4 |
|  | ec:2.5.1.18 | glutathione transferase | 6 |
|  | ec:2.5.1.16 | spermidine synthase | 2 |
|  | ec:1.8.1.7 | glutathione-disulfide reductase | 1 |
| **Glyoxylate and dicarboxylate metabolism** | ec:4.1.1.39 | ribulose-bisphosphate carboxylase | 7 |
|  | ec:4.1.3.1 | isocitrate lyase | 1 |
|  | ec:3.1.3.18 | phosphoglycolate phosphatase | 2 |
|  | ec:2.3.3.9 | malate synthase | 1 |
|  | ec:2.3.3.1 | citrate (Si)-synthase | 1 |
|  | ec:1.2.1.2 | formate dehydrogenase | 1 |
|  | ec:1.1.1.60 | 2-hydroxy-3-oxopropionate reductase | 1 |
|  | ec:1.1.1.37 | malate dehydrogenase | 7 |
|  | ec:1.1.1.29 | glycerate dehydrogenase | 1 |
|  | ec:1.1.1.26 | glyoxylate reductase | 1 |
|  | ec:4.2.1.3 | aconitate hydratase | 2 |
|  | ec:1.1.3.15 | (S)-2-hydroxy-acid oxidase | 2 |
| **Aminoacyl-tRNA biosynthesis** | ec:6.1.1.9 | valine---tRNA ligase | 2 |
|  | ec:6.1.1.6 | lysine---tRNA ligase | 2 |
|  | ec:6.1.1.5 | isoleucine---tRNA ligase | 1 |
|  | ec:6.1.1.4 | leucine---tRNA ligase | 2 |
|  | ec:6.1.1.1 | tyrosine---tRNA ligase ec:6.1.1.10 methionine---tRNA ligase | 2 |
|  | ec:6.1.1.22 | asparagine---tRNA ligase | 1 |
|  | ec:6.1.1.21 | histidine---tRNA ligase | 1 |
|  | ec:6.1.1.20 | phenylalanine---tRNA ligase | 2 |
|  | ec:6.1.1.19 | arginine---tRNA ligase | 1 |
|  | ec:6.1.1.17 | glutamate---tRNA ligase | 1 |
|  | ec:6.1.1.15 | proline---tRNA ligase | 3 |
|  | ec:6.1.1.12 | aspartate---tRNA ligase | 2 |

**Table S2.** *Selected regulatory motifs in the sequence of SlTPS5, 3 and 7 promoters analyzed by PLACE [61]*. The A of the start codon ATG was designated as +1. The promoter sequences have been described elsewhere [43].

| **Promoter** | ***Cis*- element** | **Name** | **Position (Strand)** | **Putative function** |
| --- | --- | --- | --- | --- |
| SlTPS5 | TGAC(C/T) | W-box | -1166 (-)  -688 (+)  -633 (-)  -465 (-)  -267 (-) | WRKY recognition site |
|  | CACATG | E-box  (G-box-like) | -343 (+)  -208 (-) | MYC recognition site |
|  | AACGTG | T/G-box | -1199 (-) | MYC recognition site |
|  | TACGTG | T/G-box-like | -955 (-) |  |
| SlTPS3 | CACATG | E-box  (G-box-like) | -1315 (+) | MYC recognition site |
|  | AACGTG | T/G-box | -147 (+) | MYC recognition site |
|  | TGAC(C/T) | W-box | -803 (+)  - 1682 (-)  -1151 (-)  -973 (-) | WRKY recognition site |
| SlTPS7 | AACGTG | T/G-box | -260 (+) | MYC recognition site |
|  | TGAC(C/T) | W-box | -1055 (-) | WRKY recognition site |

**Table S3.** *List of primers used*.
